# The Current Status of COVID-19 Vaccines

**DOI:** 10.3389/fgeed.2020.579297

**Published:** 2020-10-02

**Authors:** Kenneth Lundstrom

**Affiliations:** PanTherapeutics, Lutry, Switzerland

**Keywords:** inactivated, live-attenuated, protein subunit, viral vectors, DNA vaccines, mRNA-based vaccines, self-amplifying RNA

## Abstract

The current COVID-19 pandemic has substantially accelerated the demands for efficient vaccines. A wide spectrum of approaches includes live attenuated and inactivated viruses, protein subunits and peptides, viral vector-based delivery, DNA plasmids, and synthetic mRNA. Preclinical studies have demonstrated robust immune responses, reduced viral loads and protection against challenges with SARS-CoV-2 in rodents and primates. Vaccine candidates based on all delivery systems mentioned above have been subjected to clinical trials in healthy volunteers. Phase I clinical trials have demonstrated in preliminary findings good safety and tolerability. Evaluation of immune responses in a small number of individuals has demonstrated similar or superior levels of neutralizing antibodies in comparison to immunogenicity detected in COVID-19 patients. Both adenovirus- and mRNA-based vaccines have entered phase II and study protocols for phase III trials with 30,000 participants have been finalized.

## Introduction

Generally, coronaviruses such as the α-coronaviruses HCoV-229E and HCoV-NL63 and the β-coronaviruses HCoV-OC43 and HCoV have been associated with 15–30% of annual respiratory tract infections (Hamre and Procknow, 1966; Bradburne et al., 1967). Severe Acute Respiratory Syndrome (SARS), the first serious coronavirus outbreak originating in Guangdong, China, occurred in 2002–2003 and it was caused by Severe Acute Respiratory Syndrome-Coronavirus (SARS-CoV) (Anderson et al., 2004) resulting in over 8,000 recorded cases and 774 deaths (Cherry, 2004). However, SARS-CoV was modestly infectious, which contributed to it dying out in June 2003 (Peiris et al., 2003). Although Middle East Respiratory Syndrome-Coronavirus (MERS-CoV) causing the MERS outbreak in Saudi Arabia and other Middle East countries in 2012 did not spread to other regions, it still counted for 855 cases and claimed 333 deaths (Zaki et al., 2012; Aleanizy et al., 2017). Dromedary camels have been suggested as intermediate hosts (Meyer et al., 2014) as MERS-CoV replicates in camel cell lines (Eckerle et al., 2014) and an identical virus was isolated from a person who had been in contact with an infected camel and from the camel itself (Azhar et al., 2014). The limited spread of both SARS-CoV and MERS-CoV never reaching pandemic proportions has dampened the interest in developing vaccines against these coronaviruses.

The COVID-19 pandemic originating in Wuhan, China spread quickly all over the globe through person-to-person transmission, leading to the worst pandemic since the Spanish flu in 1918 (Kaplan, 2020; Yang et al., 2020). It has resulted in unprecedented medical, social and economic damage globally with more than 10 million persons infected and over 500,000 deaths by July 1, 2020 (https://www.worldometers.info/coronavirus/?#countries). The pandemic caused by the Severe Acute Respiratory Syndrome-Coronavirus 2 (SARS-CoV-2), a single-stranded (ssRNA) virus, is closely related to SARS-CoV and MERS-CoV (Lundstrom, 2020). However, SARS-CoV and SARS-CoV-2 both attach to angiotensin converting enzyme 2 (ACE2) on host cells (Li et al., 2003), while dipeptidyl-peptidase 4 (DPP4) is the host cell receptor for MERS-CoV (van Doremalen et al., 2014), an important issue related to drug and vaccine development. Furthermore, sequence analysis of SARS-CoV-2 isolated from infected patients indicated that bats, snakes, and pangolins are potential virus carriers (Yang et al., 2020). Both diagnostics and prevention of virus spread have been hampered by many carriers being asymptomatic and the variation of degree of severity of COVID-19 ranging from mild flu-like symptoms to severe pneumonia and death (Lai et al., 2020; Yang et al., 2020). Today, there are no efficient antiviral drugs or vaccines to treat COVID-19 patients and protect the general population from being infected (Pascarella et al., 2020). For these reasons, it is of outmost importance to develop urgently both antiviral drugs and vaccines. Obviously, antiviral drugs are essential for the treatment of current and future COVID-19 patients, but vaccines are absolutely necessary for allowing life to return to normal as we knew it before the pandemic hit us (Lundstrom, 2020). The focus in this review is on vaccine development, describing the different approaches from inactivated and live-attenuated viruses to protein subunit and peptide vaccines and recombinant vaccines utilizing viral expression vectors and nucleic acid-based delivery. Both preclinical studies in animal models and clinical trials conducted in humans are presented.

## Vaccine Development Strategies

**Figure 1:**
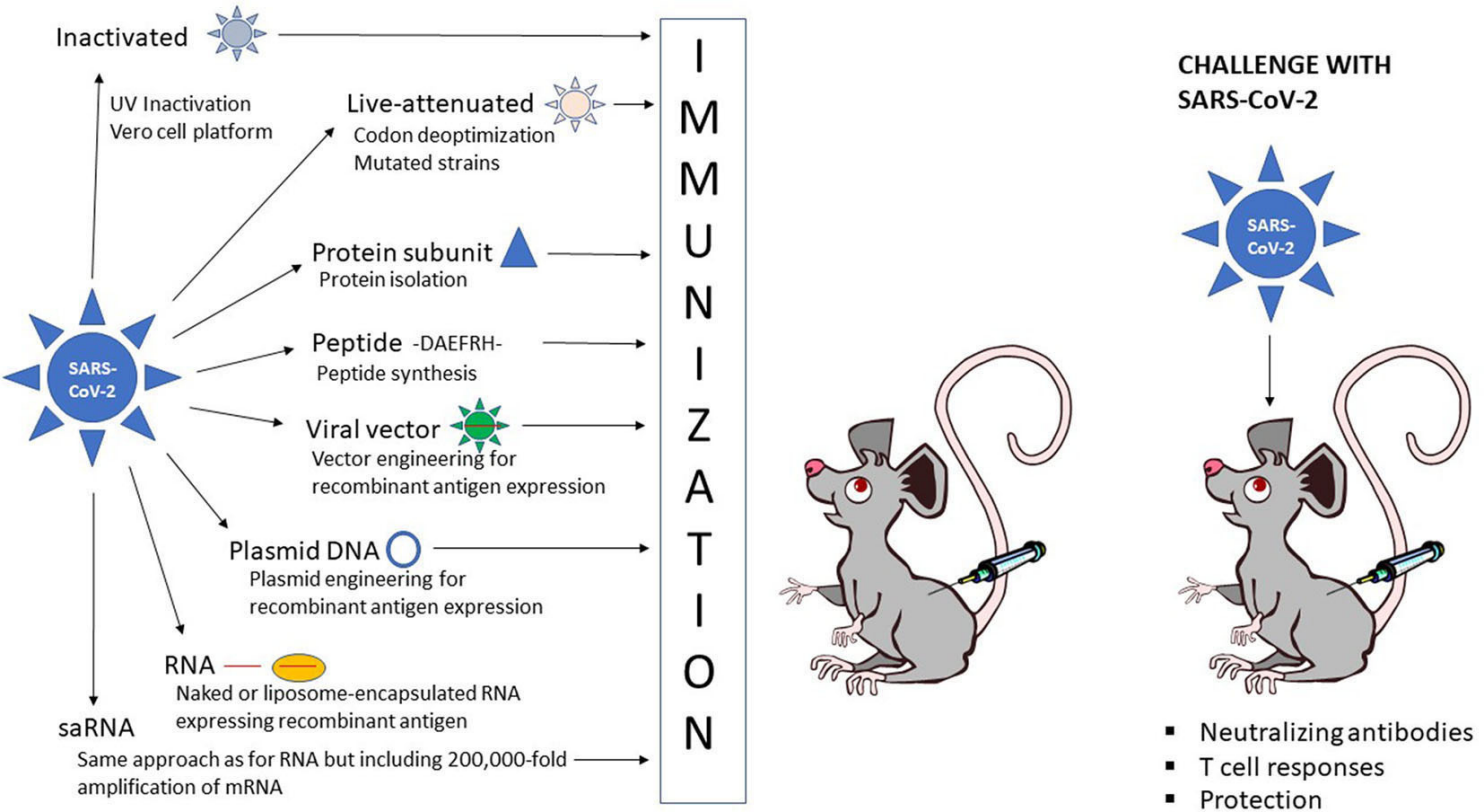
Schematic presentation of vaccine administration. Inactivated SARS-CoV-2 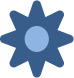, live-attenuated SARS-CoV-2 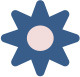, protein subunit 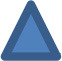, peptide, -DAEFRH- viral vector expressing SARS-CoV-2 antigen 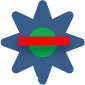, DNA plasmid expressing SARS-CoV-2 antigen 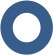, naked RNA 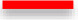 or liposome-encapsulated RNA expressing SARS-CoV-2 antigen 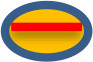.

The different strategies for vaccine development against COVID-19 can be divided into three categories: inactivated and live-attenuated viruses, protein subunit and peptide vaccines, and recombinant vaccines based on viral, DNA and RNA delivery (Figure 1). Obviously, each approach exists with its own variations and engineered modifications as described in more detail below.

### Inactivated and Live-Attenuated Vaccines

In the context of inactivated viruses, it is important that infectious viruses applied for vaccine development are completely inactivated for safety reasons, while the viral epitopes targeted for protective immunity should be conserved to enable high quality antigen production (Delrue et al., 2012). The virus inactivation process can be hampered by virus aggregate formation, protein crosslinking, denaturation and degradation, which should be addressed before vaccine administration. One approach has been to prepare UV-inactivated virus on a large scale (Tsunetsugu-Yokota, 2008). For instance, UV-inactivated SARS-CoV was prepared under Biosafety Level 3 (BSL3) regulation including virus expansion, titration, inactivation, and ultracentrifugation, which is also applicable to newly emerging viruses. A versatile Vero cell platform has been established for a wide range of vaccines, including polio virus (Barrett et al., 2017). Optimization of the platform to include a double inactivation procedure has ensured the highly robust inactivation of novel emerging viruses such as influenza virus, West Nile virus, Chikungunya virus (CHIKV), Ross River virus, SARS-CoV and Zika virus.

Related to live-attenuated viruses, they represent some of the most successful cost-effective interventions in medical history including eradication of smallpox in 1980 (Minor, 2015). Live-attenuated vaccines have functioned well for acute disease, whereas chronic infections such as HIV have been more challenging due to safety and efficacy issues. The capability of live-attenuated vaccines to elicit humoral and cell-mediated immune responses rely on their physicochemical stability (Tlaxca et al., 2015). Important factors include formulation design, types of robust dosage forms, routes of delivery and distinction between inactivated and live-attenuated vaccines. An interesting approach evaluated for the prototypic arenavirus lymphocytic choriomeningitis virus (LCMV) comprises codon deoptimization of the surface glycoprotein (GP), which demonstrated wildtype like propagation in cultured cells despite barely detectable expression of GP (Cheng et al., 2017). The codon deoptimized LCMV was highly attenuated *in vivo* but induced complete protection against lethal challenges with wildtype LCMV, showing a good safety profile and efficacy for a live-attenuated vaccine.

### Protein Subunit and Peptide Vaccines

Protein subunit vaccines date back to the time before recombinant protein expression when parainfluenza type 3 (PI-3) virus glycoproteins were isolated by their sedimentation rates after ultracentrifugation and used for immunization of mice and lambs (Morein et al., 1983). The 30S protein micelles induced high antibody responses as well as provided protection against pneumonia caused by the PI-3 virus. Since then, vaccine development has relied on recombinantly expressed protein subunits for large-scale production in sufficiently pure form for application as safe and effective vaccines (Francis, 2018). In the context of protein subunit-based vaccines, small protein domains can facilitate and stabilize protein trimerization, which has been demonstrated to enhance their immunogenicity (Morris et al., 1999). Typically, the isoleucine zipper (IZ)^3^ based on the *Saccharomyces cerevisiae* GCN4 transcriptional activator (Harbury et al., 1994) and the foldon domain (Fd) of the bacteriophage T4 fibritin protein (Güthe et al., 2004) have been widely used. However, their immunogenicity has been of concern as repeated IZ- or Fd-specific administration could lead to systemic clearance and decreased therapeutic efficacy (Baker et al., 2010). To address this problem, an IZ variant with four potential N-linked glycosylation sites (PNGS) in the heptad repeat domain were designed, which did not affect protein trimerization but induced significantly lower IZ-specific antibody responses in immunized animals when fused to two HIV-Env and influenza virus hemagglutinin (HA) antigens (Sliepen et al., 2015). Moreover, the immune response against HIV-Env and influenza virus HA were not affected. This strategy referred to as molecular clamp technology has been applied for preclinical studies on COVID-19 vaccines as described below.

In the case of peptide vaccines, it was demonstrated in the 1980s for foot-and-mouth disease virus (FMDV) that peptides from two regions of the viral protein 1 (VP1) can induce high levels of neutralizing antibodies in guinea pigs, rabbits and cattle (Bittle et al., 1982). Furthermore, a single injection protected guinea pigs from challenges with lethal doses of FMDV. The relatively small molecular size of peptides renders them poor immunogens and it requires coupling to carriers to enhance the immunogenicity (Francis, 2018). For example, FMDV peptides fused to the N-terminus of β-galactosidase have been engineered, which was known to contain several helper T cell sites for increased immune responses (Francis et al., 1987). Furthermore, it was demonstrated that vaccine candidates with a single copy of the VP1 peptide elicited only low levels of neutralizing antibodies, whereas 2–4 copies provided protection of immunized animals against challenges with FMDV (Broekhuijsen et al., 1986). In the case of proteins containing two copies, 2 mg of peptide was sufficient for achieving protection, while only 0.8 mg of the four-copy peptide was needed. Another approach involves the production of structures similar to virus-like particles (VLPs) with repeated epitopes on the surface (Clarke et al., 1987). In this context, it was demonstrated that immunogenic FMDV VP1 epitopes linked to hepatitis B virus core antigen (HBcAg) fusion particles were 100-fold more immunogenic than free disulfide dimer synthetic peptides containing B- and T-cell sites and 10 times more immunogenic than carrier-linked peptides.

### Viral Vector-Based Vaccines

Viral vectors have been commonly used as delivery vehicles for vaccines (Lundstrom, 2017). The spectrum of vectors used in vaccine development is wide including adenoviruses (Ad), lentiviruses, poxviruses, parainfluenza viruses and particularly self-amplifying RNA (saRNA) viruses such as alphaviruses, flaviviruses, rhabdoviruses, and measles viruses. The unique feature of saRNA viruses relates to the expression of the non-structural replicase genes, formation of the replicase complex and extreme RNA replication, i.e., self-amplification in the cytoplasm (Lundstrom, 2019). Depending on the polarity of the ssRNA genome of saRNA viruses, the positive strand viral RNA (alphaviruses, flaviviruses) can be directly translated in the cytoplasm or the minus strand (rhabdoviruses, measles viruses) has to be copied to a positive strand first. It is estimated that the RNA is amplified approximately 200,000 after entering the host cell cytoplasm, making saRNA virus vectors attractive for vaccine development. Another advantage of saRNA viruses relates to the flexibility of utilization of recombinant particles, RNA replicons or DNA plasmid-based vectors.

Independent of which viral vector delivery system is used, the target antigen is introduced into an appropriate expression vector, which then can be subjected to expression verification studies in cell lines and administered for *in vivo* evaluation in animal models. In any application of viral vectors, it is important to pay special attention to safety aspects and therefore addressing issues related to target of expression, potential spread of virus, duration of expression, chromosomal integration, vector immunogenicity, and adverse events related to viral delivery vector or therapeutic product. In the case of target of expression, the route of administration plays an important role as has been seen by evaluation of intramuscular, intradermal, intravenous, intranasal and oral administration using classic needle-based, needle-free and electroporation-based technologies (Zheng et al., 2018; Criscuolo et al., 2019). The spread of virus, duration of expression and chromosomal integration are strongly influenced by the type of viral vector used. Typically, vectors such as Ad and alphaviruses known to generate transient expression have been favored for immunization studies and although replication-deficient vectors have demonstrated efficacy, introduction of replication-proficient vectors has provided extended but controlled spread of antigen expression. As many viruses possess immunogenic structural proteins, efforts have been made to develop second and third generation vectors, where non-essential viral sequences have been deleted (Fukuhara et al., 2016).

### DNA-Based Vaccines

Since the development of efficient recombinant technologies, DNA-based vaccine approaches have become a reality due to the easy handling of DNA plasmids and straightforward manufacturing and stability of highly purified DNA preparations (Lee et al., 2018). The delivery of DNA vaccines can be by intramuscular injection in the form of naked plasmid DNA (Wolff et al., 1990). Alternatively, electroporation (Hooper et al., 2014), gene gun (McBurney et al., 2016), and liposome (Lundstrom and Boulikas, 2003) or polymer-based (polysaccharides and chitosan) (Sunshine et al., 2011) nanoparticles can be used. In addition, polysaccharide-based adjuvants and also chitosan can enhance the immunogenicity of vaccines (Barhate et al., 2014). Importantly, to function, DNA plasmid vectors need to be transported to the cell nucleus, which can be facilitated by engineering of nuclear localization signals into the vector (Xu et al., 2016).

In the context of gene expression from DNA vectors, the use of minicircle DNA has been demonstrated to increase therapeutic biosafety as bacterial sequences are removed from the parental plasmid leaving only the gene of interest and the promoter/terminator sequences in the construct (Darquet et al., 1997). Additionally, it has been shown that minicircles can improve the immunogenicity of DNA vaccines by inducing 10–1,000-fold increase in long-term transgene expression both *in vitro* and *in vivo* in comparison to conventional plasmid DNA (Huang et al., 2009). Moreover, studies on combinations of promoters/enhancers and post-translational regulatory elements have been conducted for optimized transgene expression (Sun et al., 2009; Shen et al., 2015). Another approach comprises codon optimization, where low-frequency eukaryotic codons in the foreign DNA are identified and replaced for high-frequency codons (Grantham et al., 1980; Nagata et al., 1999). It has been demonstrated that codon-optimized DNA vaccines can enhance immunogenicity in mice and chickens (Stachyra et al., 2016). Various synthetic biology methods have also been developed to control gene expression profiles through engineered inducers within genetic circuits, which could potentially enable the regulation of the intensity and type of immune responses (Deans et al., 2012).

Although more than 500 clinical trials have been conducted or are in progress using DNA vaccines, no DNA-based human vaccine has been approved yet (Lee et al., 2018). However. a DNA vaccine against influenza virus H5N1 in poultry has received conditional approval by the USDA (Jazayeri and Poh, 2019). In the context of human DNA vaccines, clinical trials have demonstrated that neither chromosomal integration of plasmid DNA nor development of anti-DNA antibodies occur. In attempts to improve immune responses, transfection efficiency needs to be improved using higher DNA doses (Liu and Ulmer, 2005). Moreover, prime-boost regimen with a DNA vaccine and a recombinant modified vaccinia virus Ankara (MVA) has proven useful for eliciting antibodies and T cell responses for a HIV vaccine candidate (Thompson et al., 2016). In another approach, enhanced immune responses were achieved for DNA vaccines by application of intradermal electroporation (Nilsson et al., 2015). Overall, DNA vaccines provide a potentially promising alternative for COVID-19 vaccine development as presented below for preclinical studies and clinical trials.

### RNA-Based Vaccines

In addition to DNA-based vaccines, application of RNA in the forms of mRNA and saRNA vectors has recently received increased attention (Lundstrom, 2018). In the case of mRNA-based vaccines, the immediate translation of antigens in the cytoplasm of target cells provides the means for rapid immune responses. However, mRNAs are highly sensitive to degradation due to their single-stranded structure and the presence of RNases, which has resulted in different approaches for improving RNA stability (Brawerman, 1974; Burgess, 2012). These include the incorporation of anti-reverse cap analogs (ARCAs) in the RNA, which doubled the transcription efficiency (Stepinski et al., 2001) and improved the levels and duration of protein expression in cells transfected with ARCA-capped *in vitro* transcribed RNA (Zohra et al., 2007). RNA stabilization can also be obtained by engineering the poly(A) tail at the 3′ end of mRNAs based on the synergistic interaction between poly(A) and the 5′ m7G cap sequences by binding to the poly(A) binding protein (PABP) (Bernstein et al., 1989). Poly(A) tail engineering has demonstrated that increase in length enhanced polysome formation also showing an impact on protein expression levels (Munroe and Jacobson, 1990). The optimal length of the poly(A) tail has been suggested to be between 120 and 150 nucleotides (Holtkamp et al., 2006; Mockey et al., 2006). Furthermore, 5′ and 3′ end untranslated regions (UTRs) play an important role in mRNA stability and transport from the nucleus (van der Velden and Thomas, 1999; Bashirullah et al., 2001). Chemical modification by introduction of modified pseudouridine has also resulted in improved RNA stability and enhanced translation (Kariko et al., 2005, 2008). Another approach comprises high liquid chromatography purification of *in vitro* transcribed RNA for removal of double-stranded RNA contaminants in attempts to reduce the production of type 1 interferon and pro-inflammatory cytokines (Kariko et al., 2011).

As with any other approach, delivery is a major issue of concern for RNA-based vaccines, not the least because of the stability issues related to RNA. Although naked RNA is far from optimal for delivery, intramuscular mRNA injections have demonstrated successful *in vivo* reporter gene (Wolff et al., 1990) and carcinoembryonic antigen (CEA) expression eliciting anti-CEA antibody responses in mice (Conry et al., 1995). However, the exposure of RNA to rapid degradation by ubiquitous RNases remains a problem (Probst et al., 2006; Houseley and Tollervey, 2009). Naked mRNA can also act as an adjuvant (McNamara et al., 2015), where antigen-encoding RNA elicits antigen-specific T and B cell immune responses (Pardi and Weissman, 2017). Moreover, co-stimulatory molecules like CD40L when co-expressed with mRNA can further enhance the immunogenicity (Schlake et al., 2012). To improve and facilitate mRNA delivery and penetration of cell membranes, gene gun-based systems including mRNA coated in gold particles have been applied (Dileo et al., 2003). In the context of melanoma, mRNA expressing the melanocyte self-antigen TRP2 fused to EGFP was delivered by gene gun, which led to antigen-specific humoral and cellular immune responses and protection against B16 melanoma lung metastasis in a mouse model (Steitz et al., 2006). Protamine condensation of mRNA has also been demonstrated to provide protection against RNA degradation (Sköld et al., 2015) and can further stimulate antigen-specific IgG antibodies and activate specific cytotoxic T lymphocyte (CTL) responses (Hoerr et al., 2000). Obviously, encapsulation has provided improved means for stability and delivery of RNA by application of liposomes and biopolymers. For instance, the cationic liposome DOTAP can provide protection against nuclease degradation and enhance cellular uptake (Phua et al., 2013). Intradermal administration of DOTAP liposomes into mouse ear pinna, protected animals against subcutaneous tumor challenges with EG7-OVA cells (Hess et al., 2006). Moreover, DOTAP/DOPE formulations showed fusogenic properties, which resulted in enhanced CTL responses. The important role of the professional antigen presenting dendritic cells (DCs) in stimulation of immune responses has also accelerated the formulation of nanoparticles optimized for targeting DCs (Ahmad et al., 2017). Immunization with DCs transfected with mRNA expressing CEA showed good tolerance in pancreatic cancer patients in a phase I study resulting in a complete response in one patient, minor responses in two individuals and stable disease in three patients with progressive disease in the remaining 18 patients (Morse et al., 2003). Moreover, DCs transfected with mannosylated, histidylated lipoplex nanoparticles expressing tumor antigen mRNA showed enhanced inhibition of B16F10 melanoma growth and prolonged survival in mice (Perche et al., 2011).

Finally, in the context of RNA-based vaccines, application of saRNAs has opened new possibilities. The cytoplasmic RNA amplification generates superior quantities of mRNA providing improved immune responses with the requirement of significantly lower doses of RNA (Lundstrom, 2019). Although naked saRNA has been demonstrated to provide good immune responses, formulations with different lipids such as ionizable C12-200 and cationic DDA and DOTAP generated lipid nanoparticles (LNPs) with exterior or interior saRNAs (Blakney et al., 2019). The LNPs formulated with cationic lipids protected saRNA from degradation even when the saRNA was absorbed to the surface. Both saRNA LNPs encapsulated with cationic and ionizable lipids showed transfer *in vitro* and *in vivo* and induced equivalent antibody responses against HIV-1 Env gp140.

## Preclinical Studies

### Inactivated and Live-Attenuated Vaccines

The various approaches for vaccine development described above have been evaluated for COVID-19 vaccines in preclinical studies (Table 1). For instance, an inactivated SARS-CoV-2 vaccine candidate developed at the Beijing Institute of Biological Products induced high levels of neutralizing antibodies in mice, rats, guinea pigs, rabbits, and non-human primates (Wang et al., 2020). Moreover, two doses of 2 mg of inactivated SARS-CoV-2 provided protection against intratracheal challenges in rhesus macaques with no detectable antibody-dependent increase of infection. In another study. a pilot-scale purified inactivated SARS-Cov-2 vaccine candidate induced SARS-CoV-2 specific neutralizing antibodies in mice, rats, and non-human primates (Gao et al., 2020). It was also demonstrated that induced antibodies neutralized 10 representative SARS-CoV-2 strains, indicating a broad neutralizing activity. Partial or complete protection against SARS-CoV-2 was observed in macaques after three immunizations with 3 or 6 μg per dose, respectively.

**Table 1:** Examples of preclinical studies on COVID-19 vaccines.

**Vaccine**	**Approach**	**Response**	**References**
Inactivated virus	Inactivated SARS-CoV-2	Protection in rhesus macaques Partial/complete protection in macaques	Wang et al., 2020; Gao et al., 2020
Live-attenuated virus	Codon deoptimized SARS-CoV-2 vaccine	Safety and efficacy studies in progress in animal models	www.scienceboard.net
Protein subunit	Yeast expressed SARS- CoV RBD CLPs for SARS-CoV-2 Drosophila S2 VLPs Molecular clamp CoV-2 MNA SARS-CoV-2 S1 SARS-CoV-2 S protein subunit trimeric vaccine Baculovirus-based SARS- CoV-2 S expression	Protection against SARS-CoV in mice, could be developed for COVID-19 SARS-CoV-2 nAbs in mice SARS-CoV-2 nAbs in mice High levels of nAbs Robust nAb responses in mice Cross-reactive Abs from sera from COVID-19 patients Preclinical studies in progress	Chen et al., 2020 https://www.adaptvac.com/ www.expreS2ionbio.com www.csl.com/news Kim et al., 2020 www.cloverbiopharma.com www.news.sanofi.us
Peptide	Computer designed SARS- CoV-2 E peptide vaccines DPX-SARS CoV-2 SARS-CoV-2 epitopes in microspheres	10 potential vaccine candidates to be tested *in vivo* Robust antibody responses *in vitro* Preclinical studies in primates	Abdelmageed et al., 2020 www.imv-inc-com www.flowpharma.com
Viral vector	Ad SARS-CoV-2 S Ad5 SARS-CoV-2 S Ad26.COV2-S Simian Ad SARS-CoV-2 MVA-CoV-2 VLPs Replicating MV-CoV-2 PIV5-SARS-CoV-2 S	Prevention of pneumonia in macaques Good safety profile, robust immune responses in animal models Strong immune responses in animals Preclinical studies in progress Preclinical studies in progress Preclinical studies in progress Preclinical studies in progress	van Doremalen et al., 2020 www.thepharmaletter.com www.janssen.com www.reithera.com/ www.geovax.com www.themisbio.com https://research.uga.edu/covid/
DNA	DNA SARS-CoV-2 S EP DNA SARS-CoV-2 EP DNA SARS-CoV-2 Needle-free DNA SARS- CoV-2 bacTRL-Spike (SARS- CoV-2 S)	Antigen-specific T cell responses, Inhibition of SARS-CoV-2 infection nAbs in immunized primates Preclinical studies in progress Preclinical studies in progress Preclinical studies on probiotic bac-terial vaccine production in the gut	Smith et al., 2020 www.pulsenews.co.kr https://ki.se/en/research/opencorona https://www.abnova.com https://www.symvivo.com/covid-19
RNA	Prefusion-stabilized LNP mRNA CoV-2 S LNP mRNA SARS-CoV-2 LNP mRNA CoV-2 S LUNAR® mRNA SARS CoV-2 delivery Intranasal mRNA for con- served CoV-2 regions STARR™ saRNA using LUNAR® delivery LNP saRNAs	nAbs and CD8+ T cell responses, mice protected against SARS-CoV-2 High level nAbs in animal models Robust Ab response in animal models Positive immunogenicity in preclinical studies Preclinical studies in progress Enhanced seroconversion and IgG and IgM antibody titers Preclinical studies in progress	Corbett et al., 2020 www.curevac.com/covid-19 https://www.daiichisankyo.com https://arcturusrx.com/ https://epivax.com https://arcturusrx.com/ www.imperial.ac.uk

*Ad, adenovirus; bacTRL-Spike, probiotic bacterial SARS-CoV-2 S in the gut; CLPs, capsid-like particles; DPX, platform based on nanoscale lipid particles; EP, electroporation; LNP, liposome nanoparticles; LUNAR®, Lipid-enabled and Unlocked Nucleomonomer Agent modified RNA; MNA, Microneedle array; MV, Measles virus; MVA, Modified Vaccinia Ankara; nAbs, neutralizing antibodies; RBD, receptor-binding domain; S2, Drosophila Schneider 2 cells; VLPs, virus-like particles*.

Although designer vaccines have to some extent replaced live-attenuated vaccines due to safety issues, determination of naturally circulating attenuated SARS-CoV-2 variants is of importance (Armengaud et al., 2020). Identification of existing attenuated SARS-CoV-2 variants is instrumental not only to better understand the evolution of SARS-CoV-2, but also to provide the means of predicting the dynamics of the current and possible future pandemics (Ng et al., 2003). In the context of live-attenuated virus-based vaccines, codon deoptimization as described for LCMV (Cheng et al., 2017) has been applied for SARS-CoV-2. Codagenix in collaboration with the Serum Institute of India has successfully synthesized a codon deoptimization-based live-attenuated vaccine candidate called CDX-005, which is currently undergoing safety and efficacy studies in animal models (www.scienceboard.net).

### Protein Subunit Vaccines

Protein subunit vaccines received a major boost with the introduction of genetic engineering technologies allowing rapid large-scale expression and purification of recombinant proteins to be applied for immunizations. In this context, the SARS-CoV receptor-binding domain (RBD) was expressed in *Pichia pastoris* yeast as protein RBD219-N1 and manufactured and purified under cGMP conditions (Chen et al., 2014), which showed high-levels of neutralizing antibodies and protective immunity in mice against challenges with a homologous SARS-CoV MA15 strain. As the overall amino acid similarity between SARS-CoV and SARS-CoV-2 S and RBD domains is high and convalescent serum from SARS-CoV patients can neutralize SARS-CoV-2, RBD219-N1 could be repurposed as a heterologous vaccine against COVID-19 (Chen et al., 2020). However, the big conformational differences of S proteins between SARS-CoV and SARS-CoV-2 might prevent success. One approach for protein subunit vaccines has involved capsid-like particles (CLPs), which are multimeric, repetitive assemblies of recombinant viral capsid proteins (Aves et al., 2020). CLPs are highly immunogenic and can be used as molecular scaffolds for soluble vaccine antigens and provide a versatile and efficient technology. AdaptVac and the PREVENT-nCoV consortium have utilized CLPs for a COVID-19 vaccine, which induced high levels of neutralizing antibodies in mice and prevented SARS-CoV virus from infecting human cells *in vitro* (https://www.adaptvac.com/news). Moreover, ExpreS2ion announced that using their VLP technology based on Drosophila S2 cells, generated very high levels of SARS-CoV-2 neutralizing antibodies in a mouse model (https://expres2ionbio.com/investor/international-press-releases/). In another approach, molecular clamp technologies have improved the immunogenicity by locking of unstable prefusion versions (Sliepen et al., 2015) resulting in robust generation of neutralizing antibodies against SARS-CoV-2 (https://www.csl.com/news/2020/20200605-uq-cepi-and-csl-partner-for-covid-19-vaccine-candidate). Based on codon optimized MERS-CoV S1 subunit vaccines fused with a foldon trimerization domain (Xiao et al., 2004), a microneedle array (MNA)-delivered subunit vaccine elicited strong and long-lasting antigen-specific antibody responses *in vivo* (Kim et al., 2020). Furthermore, a clinically translatable MNA-SARS-CoV-2 vaccine was designed and produced, which showed potent antigen-specific antibody responses in mice (Kim et al., 2020). In another approach, SARS-CoV-2 subunit trimer vaccines with the native-like trimeric structure and antigenic epitopes of S protein were designed and were submitted to expression in mammalian cells, which detected high-titer cross-reactive antibodies from previously infected COVID-19 patients (http://www.cloverbiopharma.com/index.php?m=content&c=index&a=lists&catid=42). Related to protein subunit vaccines, a full-length and a truncated version containing the ecto-domain of SARS-CoV S protein were expressed from a baculovirus vector in insect cells (Zhou et al., 2006). Both purified proteins elicited strong immune responses in immunized mice. Baculovirus-based expression has been applied for the SARS-CoV-2 S protein to develop a COVID-19 vaccine, which is now at the preclinical stage (http://www.news.sanofi.us/press-releases?item=137254).

### Peptide Vaccines

In a combination of immune-informatics and comparative genomics, the SARS-CoV-2 envelope protein (E) was used for the design of T-cell epitope-based peptide vaccines (Abdelmageed et al., 2020). Ten promising peptide vaccine candidates binding to MHC class I and MHC class II were identified with a 88.5 and 99.99% global population coverage, respectively. Next, the vaccine candidates need to be tested in animal models. In another approach, a nine amino acid long peptide NP44-52 (YQVNNLEEI) from the conserved region of Ebola virus (EBOV) nucleocapsid protein (NP) provided protection against EBOV challenges after a single immunization of C57BL/6 mice (Herst et al., 2020). This approach should also be feasible for SARS-CoV-2 as its nucleocapsid proteins contain multiple Class I epitopes with predicted HLA restrictions. The peptide based DPX vaccine based on nanoscale lipid particles containing an antigen and an adjuvant demonstrated robust antibody responses against respiratory syncytial virus (RSV) in a phase I trial (Torrey et al., 2020). It has triggered the design of non-overlapping neutralizing epitopes, which can potentially act synergistically on different key mechanism actions of the SARS-CoV-2 S protein (https://imv-inc.com/clinical-trials/dpx-covid-19/). A total of 23 peptides formulated applying the DPX platform showed robust antibody responses after the first or second immunization in mice and an optimal peptide combination was selected for targeting of the attachment, fusion and entry of SARS-CoV-2 into human cells (https://www.businesswire.com/news/home/20200521005173/en/IMV-Announces-Selection-of-a-Vaccine-Candidate-Against-COVID-19-to-Advance-Into-Human-Clinical-Studies). The peptide combination elicited equivalent or superior antibody responses compared to the DPX-RSV peptide epitope vaccine. In another peptide-based vaccine development program, synthetic biodegradable PLGA microsphere-based vectors containing two HIV epitopes elicited CTL responses in C57BL/6 mice (Rubsamen et al., 2014). Based on these findings, FlowVax microspheres have been employed for the design of a COVID-19 vaccine targeting such SARS-CoV-2 epitopes, which are least likely to mutate. The vaccine candidates are currently evaluated in a primate model (www.flowpharma.com).

### Viral Vector-Based Vaccines

As non-replicating viral vectors present a popular approach for vaccine development for infectious diseases it is no surprise that numerous preclinical studies for COVID-19 vaccine candidates have been conducted. In this context, expression of the SARS-CoV-2 S protein from the simian adenovirus vector ChAdOx1-S (Folegatti et al., 2019) elicited strong humoral and cellular immune responses in mice and rhesus macaques (van Doremalen et al., 2020). Furthermore, it was demonstrated that SARS-CoV-2 pneumonia was prevented in macaques immunized with ChAdOx1-S. In another study, adenovirus 5 (Ad5) expressing the SARS-CoV-2 S protein showed a good safety profile with only minor adverse events such as pain at the injection site and fever responses and induced strong immune responses in animal models (https://www.thepharmaletter.com/article/cansino-s-covid-19-vaccine-promising-but-more-research-needed). The adenovirus type 26 (Ad26) vector, previously applied for EBOV vaccine development (Ad26.ZEBOV) (Mutua et al., 2019), has more recently been designed as Ad26.COV2-S for a COVID-19 vaccine candidate, which has shown promising results in preclinical trials eliciting strong immune responses in animal models (https://www.janssen.com). In another study, a chimpanzee adenovirus vector (Vitelli et al., 2017) has been employed for preclinical studies for a single-dose COVID-19 vaccine (https://www.reithera.com/). Poxviruses, especially vaccinia viruses such as the Modified Vaccinia Ankara (MVA) strain has been successfully applied for vaccine development against EBOV (Domi et al., 2018) and Lassa virus (Salvato et al., 2018), which has also encouraged their use for COVID-19 vaccines. In this context, a non-replicating MVA-based VLP vaccine for COVID-19 has entered preclinical evaluation (https://www.geovax.com/technology-pipeline/infectious-diseases). Moreover, live-attenuated replicating measles virus (MV) vectors, previously shown to protect mice from lethal challenges with CHIKV (Brandler et al., 2013) also demonstrated good safety, tolerability and immunogenicity in a phase II trial (Reisinger et al., 2019). Recently MV vectors have also been subjected to COVID-19 vaccine development (https://www.themisbio.com/our-programs/pipeline/). Another viral vector system based on the parainfluenza virus 5 (PIV5) has been applied for MERS vaccine development (Li et al., 2020). It was demonstrated that a single dose of PIV5 expressing the MERS-CoV S protein induced neutralizing antibodies and T cell responses and protected intranasally immunized mice from challenges with lethal doses of MERS-CoV. Recently, PIV5-based vaccine development has been expanded to COVID-19 (https://research.uga.edu/covid/). In another approach, findings that SARS-CoV-2 infections have been associated with potentially severe neurological symptoms, similar to those caused by rabies virus (RABV), has triggered the interest in using RABV vectors in vaccine development to selectively target and neutralize CNS penetration (Stefano et al., 2020). Non-replicating RABV vectors have demonstrated high levels of expression of chimeric capsid proteins containing HIV-1, MERS-CoV, EBOV and hepatitis C virus sequences. Non-replicating RABV vectors expressing chimeric capsid proteins with discrete SARS-CoV-2 S protein domains, should therefore provide a highly efficient preventive approach against neurological comorbidities of COVID-19.

### DNA-Based Vaccines

DNA vaccines have been subjected to numerous preclinical studies. For instance, a DNA plasmid expressing the SARS-CoV-2 S protein provided robust expression in cell lines and elicited antigen-specific T cell responses and functional antibodies in immunized mice and guinea pigs (Smith et al., 2020). The functional antibodies neutralized SARS-CoV-2 infection and blocked S protein binding to the ACE2 receptor. Biodistribution studies indicated a statistically significant increase in SARS-CoV-2 S protein binding IgG in both BALB/c mice and guinea pigs, demonstrating the presence of anti-SARS-CoV-2 specific antibodies in the lungs. In another study, electroporation-enhanced DNA immunization showed targeting of HPV antigens to dendritic cells, leading to E6/E7-specific IFN-γ-producing T cell responses in patients with cervical intraepithelial neoplasia 3 (CIN3) and complete regression of lesions in seven out of nine patients (Kim et al., 2014). These promising findings have encouraged the application of electroporation-enhanced DNA immunization for COVID-19 vaccines resulting in induction of neutralizing antibodies in immunized primates (www.pulsenews.co.kr). Another DNA vaccine approach is based on the expression of different forms of SARS-CoV-2 leading to humoral and cellular immune responses in rhesus macaques (Yu et al., 2020). Moreover, immunized primates were challenged with SARS-CoV-2 showing significant decrease in viral loads in bronchoalveolar lavage and nasal mucosa. A correlation between vaccine-elicited neutralizing antibody titers and protective efficacy was observed, indicating that vaccine protection correlated with the vaccine immunogenicity. In another DNA vaccine approach, several chimeric SARS-CoV-2 genes delivered by *in vivo* electroporation will be evaluated for the most potent DNA immunotherapy candidate, which can provide protection against SARS-CoV-2 (https://ki.se/en/research/opencorona). The DNAx™ Immune technology has been combined with a needle-free technology for the development of DNA-based COVID-19 vaccines (https://www.abnova.com), which is currently in the preclinical phase. An interesting approach comprises the bacTRL platform, in which genetically modified probiotic bacteria are orally administered for the colonization of the gut, where they bind directly to epithelial cells (Ding et al., 2018). There, they constitutively replicate and express antigens from DNA plasmids generating neutralizing nanobodies, consisting of the variable VHH domains of camelid antibodies (Huo et al., 2020). Robust mucosal and systemic humoral and cell-mediated immunity can be established in lymphoid tissues and the expression of neutralizing nanobodies provides immediate passive immunity. This approach of a living-medicine supplies vaccine production throughout the life of the bacterial colony. Currently, the platform has been utilized for preclinical testing of a bifidobacterial monovalent SARS-CoV-2 DNA vaccine (bacTRL-Spike) for rapid induction of cellular and humoral immunity against SARS-CoV-2 S and prevention of COVID-19 infection (https://www.symvivo.com/covid-19). Moreover, a trivalent vaccine of SARS-CoV-2 S, nucleocapsid and matrix glycoprotein (bacTRL-Tri) is under development. Several other DNA-based vaccine studies are at the preclinical stage, for which information can be found on the continuously updated https://www.who.int/publications/m/item/draft-landscape-of-covid-19-candidate-vaccines website.

### RNA-Based Vaccines

RNA vaccines in the form of mRNA, liposome encapsulated mRNA and self-amplifying (saRNA) have been subjected to preclinical evaluation. The expression and immunogenicity of SARS-CoV-2 spike trimers has been optimized by prefusion-stabilizing mutations resulting in the mRNA-1273 vaccine, which formulated in LNPs induced both potent neutralizing antibody and CD8^+^ T cell responses and protected immunized mice against SARS-CoV-2 infection (Corbett et al., 2020). In another study, mRNA has been customized in the 5′ and 3′ end untranslated regions and in the open reading frame to ensure translation of ideal levels of protein (https://www.curevac.com/mrna-platform). LNP mRNA-based delivery has previously been demonstrated to provide protection against CHIKV challenges in immunized mice (Kose et al., 2020) and has been applied for COVID-19 vaccine development by engineering several SARS-CoV-2 constructs (https://www.curevac.com/covid-19). The lead vaccine candidate elicited high levels of neutralizing titers in animal models. Another COVID-19 vaccine development program has been initiated with LNP mRNA-based delivery, which has generated robust antibody responses in animal models (https://www.daiichisankyo.com). Moreover, a multi-component delivery system called Lipid-enabled and Unlocked Nucleomonomer Agent modified RNA (LUNAR®) with access to over 150 proprietary lipids has been utilized for mRNA-based COVID-19 vaccines, which has generated positive immunogenicity data in preclinical studies (https://arcturusrx.com/). In another approach, the mRNA TriMix platform was used for preclinical development of COVID-19 vaccines (https://epivax.com). The strategy comprises the selection of conserved epitopes from the whole SARS-CoV-2 genome eliciting strong cellular T cell-based responses based on *in silico* epitope predictions, which will also potentially provide protection against future variations of the virus. The Trimix technology contains an mRNA-based vaccine adjuvant, which stimulates DCs into activating strong CD4 and CD8 T cell responses. The intranasal delivery supports primary immune defense and memory responses against viral replication and colonization of the lungs.

### Self-Amplifying RNA-Based Vaccines

In the context of saRNA, a few preclinical studies have been initiated. For instance, the self-replicating RNA system STARR™ mRNA has demonstrated superiority over conventional mRNA approaches (https://arcturusrx.com/). A single dose of STARR™ saRNA with LUNAR® based delivery induced significantly higher seroconversion relative to conventional mRNA and the anti-SARS-CoV-2 IgG and IgM antibody titers were also higher. Moreover, in another COVID-19 vaccine development study, LNPs formulated with exterior and interior saRNAs as described above (Blakney et al., 2019), have demonstrated that 1,000 times lower RNA doses can be used compared to conventional mRNA to achieve the same immune responses. Preclinical evaluation has demonstrated a good safety profile and encouraging signs of effective immune responses (https://www.imperial.ac.uk/mrc-global-infectious-disease-analysis/covid-19/).

## Clinical Trials

The number of clinical trials including vaccines based on inactivated viruses, protein subunits, viral vector-based delivery and nucleic acids are continuously increasing with more than 20 trials conducted, in progress or for which study protocols have been finalized as indicated by the WHO reporting of June 29, 2020 (https://www.who.int/publications/m/item/draft-landscape-of-covid-19-candidate-vaccines) (Table 2). In the context of inactivated virus, a randomized, double-blind, placebo parallel-controlled phase I/II clinical trial for an inactivated COVID-19 vaccine produced in Vero cells has been planned for healthy volunteers aged 6 years and older for the evaluation of safety and immunogenicity (ChiCTR2000031809). Recruitment is also on-going for another Chinese phase I/II study with inactivated SARS-CoV-2 vaccine produced in Vero cells (ChCTR2000032459). In the randomized, double-blind, placebo parallel-controlled study the evaluation of safety and immunogenicity will take place in healthy volunteers after immunization with different doses. Although recruitement is still in progress it has been reported that more than 2,000 people have been vaccinated showing good safety profiles (http://www.xinhuanet.com/english/2020-06/01/c_139105909.htm). These two phase I/II trials are anticipated to be completed by the end of the year. In another randomized, double-blinded, single-center, placebo-controlled phase I/II trial with a purified inactivated SARS-CoV-2 virus vaccine candidate, 422 healthy subjects age 60 or older will be evaluated for safety and immunogenicity (NCT04383574). Seventy-two patients are recruited for phase I and 350 volunteers for phase II. Another similar phase I/II study will comprise 144 healthy volunteers in phase I and 600 in phase II in the age group 18–59 years, who will receive two doses of the inactivated COVID-19 virus vaccine candidate or placebo on days 0 and 14 or days 0 and 28 (NCT04352608). Furthermore, a randomized, double-blind and placebo-controlled phase Ia/IIa trial in 18–59 years old healthy volunteers will enroll 192 and 750 individuals for phase Ia and phase IIa, respectively (NCT04412538). In phase Ia, two immunizations with low, medium or high doses of inactivated COVID-19 vaccine candidate or placebo are scheduled at days 14 or 28, while medium and high doses are planned for the phase IIa study at days 14 or 28.

**Table 2:** Clinical trials for COVID-19 vaccines.

**Vaccine type**	**Affiliation**	**Stage**	**Findings**	**References**
Inactivated virus	Wuhan Institute of Biological Products, Sinopharm	Phase I/II	Patient recruitment in progress	ChiCTR2000031809
Inactivated virus	Beijing Institute of Biological Products, Sinopharm	Phase I/II	Patient recruitment in progress Good safety profiles observed in vaccines	ChCTR2000032459
Inactivated virus + alum	Sinovac	Phase I/II	Recruitment of 72 volunteers for phase I, 350 for phase II	NCT04383574
Inactivated virus + alum	Sinovac	Phase I/II	Recruitment of 144 volunteers for phase I, 600 for phase II	NCT04352608
Inactivated virus	Institute of Medical Biology, Chinese Academy of Medical Sciences	Phase Ia/IIa	Phase Ia: 192 patients, low, medium or high dose Phase IIa: 750 patients, medium or high dose	NCT04412538
Protein subunit CoV-2 S + Adj	Novavax	Phase I/II	Phase I data on safety and immunogenicity before phase II	NCT04368988
Protein subunit	Clover Biopharmaceuticals, GSK, Dynavax	Phase I	High-titer cross-reacting Abs in 100% of sera from COVID-19 patients; Trial in progress on safety and immunogenicity	https://www.dynavax.com NCT04405908
Protein subunit CoV-2 S RBD-dimer	Anhui Zhifei Longcom Biopharmaceuticals	Phase I	Phase I trial confirmed	https://www.reuters.com/article/health-coronavirus-zhifei-vaccine-idUSL4N2EH16C
Viral Vector Ad26.COV2-S	Janssen Pharmaceuticals	Phase I/II	Phase I trial to start in July 2020	https://www.janssen.com
Viral Vector Ad Gam-COVID-Vac	Gamaleya Research Institute	Phase I	Prime-boost phase I trial in progress	CT04436471
Viral Vector Ad Gam-COVID-Vac Lyo	Gamaleya Research Institute	Phase I	Prime-boost phase I trial in progress	CT04437875
Viral Vector Ad5 SARS-CoV-2 S	CanSino Biological Inc., Beijing Institute of Biotechnology	Phase I	Safe, tolerable vaccination, nAbs responses	Zhu et al., 2020 ChiCTR2000031781
Viral Vector Ad5 SARS-CoV-2 S	CanSino Biological Inc., Beijing Institute of Biotechnology	Phase II	Study in progress	ChiCTR2000030906
Viral Vector ChAdOx1-S	University of Oxford	Phase I/II	Phase I in progress with >1000 immunizations done	2020-001072-15
Viral Vector ChAdOx1-S	University of Oxford, Clinics in South Africa	Phase I/II	Recruitment of 2000 individuals in progress	PCTR202006922165132
Viral Vector ChAdOx1-S	University of Oxford	Phase IIb/III	Recruitment of patients in progress	2020-001228-32
Viral Vector ChAdOx1-S	University of Oxford, Univ. Sao Paulo, Brazil	Phase III	Recruitment of patients in progress	ISRCTN89951424
Viral Vector LV-SMENP DC	Shenzhen Geno-Immune Medical Institute	Phase I/II	Recruitment of patients in progress	NCT04276896
DNA GX19 + EP	Genexine Consortium	Phase I/II	Recruitment of patients in progress	NCT04445389
DNA INO-4800 + EP	Inovio Pharmaceuticals	Phase I	Study in progress, plans for PhaseII/III trial	NCT04336410
LNP mRNA SARS-CoV-2	Curevac	Phase I	Recruitment of patients in progress	www.curevac.com/covid-19
LNP uRNA, modRNA and saRNA CoV-2	BioNTech, Fosun Pharma, Pfizer	Phase I/II	Phase I trial in progress 1.8-2.8 increase in nAbs titers compared to COVID-19 convalescent sera	NCT04368728 Mulligan et al., 2020
LNP mRNA SARS-CoV-2 S	Moderna, BARDA	Phase I	Initial results of elevated nAb titers in 8 tested individuals	NCT04283461https://investors.modernatx.com
LNP mRNA SARS-CoV-2 S	Moderna, BARDA	Phase II/III	Phase II study in progress, Phase III trial planned	NCT044
LNP saRNA SARS-CoV-2 S	Imperial College London, Medical Research Council	Phase I/II	Study in progress in 18-45 years old volunteers	ISRCTN17072692

*Abs, antibodies; Adj, adjuvant, BARDA, Biological Advanced Research and Development Authority; DC, dendritic cell; EP, electroporation; LV, lentivirus; LNP, liposome nanoparticles; modRNA, nucleoside modified mRNA; saRNA, self-amplifying RNA; uRNA, uracil containing mRNA*.

In the case of protein subunit-based vaccines, a two-part phase I/II randomized, observer-blinded, placebo-controlled trial has been designed to evaluate the safety and immunogenicity of a SARS-CoV-2 S nanoparticle vaccine with or without Matrix-M™ adjuvant (NCT04368988). An estimated 131 healthy volunteers of 18–59 years of age will be recruited and in the first part of the study, two SARS-CoV-2 S constructs will be evaluated in two cohorts (https://ir.novavax.com/news-releases/news-release-details/novavax-initiates-phase-12-clinical-trial-covid-19-vaccine). Interim analysis of safety and immunogenicity data will provide insight into the possible expansion to the second part of the trial in multiple countries including the US for the assessment of safety, immunity and reduction of COVID-19 in a broader age range. In another approach, a SARS-CoV-2 Spike Protein Subunit-Trimer vaccine has demonstrated high-titer cross-reacting antibodies in 100% of sera from 11 previously infected COVID-19 patients in China (https://www.dynavax.com/COVID-19_information/). It was also confirmed that the native-like structure of the S protein was preserved by the S-Trimer. In a randomized, double-blind, placebo-controlled, first-in-human phase I trial the safety, reactogenicity and immunogenicity at multiple dose levels will be evaluated with or without an adjuvant in healthy volunteers (NCT04405908). Recently, a new COVID-19 vaccine candidate based on the SARS-CoV-2 S RBD Dimer has been approved in China for a phase I clinical trial (http://en.nhc.gov.cn/2020-06/24/c_80896.htm).

In the context of viral vector-based vaccines, the encouraging results from preclinical studies have accelerated the initiation of a randomized, double-blind, placebo-controlled phase I/II trial in 1045 healthy volunteers in the age groups of 18–55 years and older than 65 years in Belgium and the US with the Ad26.COV2-S recombinant vector (https://www.janssen.com). The study is expected to start in the second half of July 2020. In another approach, a combination of Ad5 and Ad26 vectors has been engineered to express SARS-CoV-2 genes and will be subjected to a non-randomized phase I clinical trials. The Gam-COVID-Vac will be tested in healthy volunteers by intramuscular administration for safety, tolerability, and immunogenicity in a prime-boost immunization regimen (NCT04436471). In stage 1, 18 volunteers will be immunized and monitored for 5 days. The second stage includes 20 volunteers, who will be subjected to a booster scheme. A similar non-randomized phase I trial will be conducted with a lyophilized version of the Ad virus vector-based drug, namely Gam-COVID-Vac Lyo (NCT04437875). In stage 1, 18 volunteers will receive a single intramuscular injection and will be monitored for 5 days. As above, 20 volunteers will receive a second booster immunization. The first-in-human, dose-escalation, open-label, non-randomized phase I study with Ad5 expressing the SARS-CoV-2 S protein was conducted in China (ChiCTR2000030906; Zhu et al., 2020). Three doses of 5 × 10^10^, 1 × 10^11^, and 1.5 × 10^11^ viral particles were administered intramuscularly in 108 healthy 18–60 years old volunteers and the safety, tolerability and immunogenicity were monitored. Some mild to moderate injection site pain was detected but no serious adverse events were observed. Immunization elicited neutralizing antibodies with a peak humoral response and rapid specific T cell responses 28 and 14 days after vaccination, respectively. The Ad5-based vaccine candidate has entered a phase II trial from which additional information on safety and immunogenicity is expected shortly (ChiCTR2000030906). The simian adenovirus vector ChAdOx1, which has elicited strong humoral and cellular immune responses in mice and rhesus macaques (van Doremalen et al., 2020), has been subjected to a phase I/II clinical trial to evaluate the safety and immunogenicity in healthy volunteers in the UK (2020-001072-15). Today, more than 1,000 immunizations have been executed and follow-up is in progress (https://covid19vaccinetrial.co.uk/ongoing-studies). Moreover, the safety, immunogenicity and efficacy will be assessed in a double-blinded, placebo-controlled, individually randomized trial in 18–65 years old individuals with or without HIV in South Africa (PACTR202006922165132). The vaccine candidate will be intramuscularly administrated to 1,950 HIV-uninfected and 50 people living with HIV and will be followed up for 12 months. Moreover, a phase IIb/III study for the ChAdOx1-S vaccine candidate has been initiated with the aim at enrolling up to 10,260 adults and children (2020-001228-32). In another phase III trial, ChAdOx1-S will be administered to 2,000 healthy volunteers in the age group 18–55 years in Brazil and will be followed for 12 months. Finally, a lentivirus-based phase I/II trial in 100 healthy volunteers will be conducted in Shenzhen, China (NCT04276896). The lentivirus-based vaccine contains minigenes of multiple conserved regions of SARS-CoV-2 transduced into DCs. After subcutaneous injection of 5 × 10^6^ cells of LV-DC combined with intravenous administration of 1 × 10^8^ antigen-specific CTLs, the safety and immunogenicity will be evaluated.

DNA-based vaccines have also entered the clinical phase. A dose-escalation, single arm, open-labeled phase I/II study has been planned for the intramuscularly EP-aided delivery of the GX-19 DNA vaccine expressing SARS-CoV-2 genes (NCT04445389). In phase I, a total of 40 healthy volunteers will be enrolled and the randomized, double-blind, placebo-controlled phase II follow-up study is planned to comprise a total of 150 individuals. Previously, it was demonstrated in a dose-escalation phase I trial that intramuscular injection immediately followed by co-localized EP of plasmid DNA encoding MERS-CoV sequences was well-tolerated with no vaccine-related serious adverse events and immune responses in more than 85% of participants after two vaccinations (Modjarrad et al., 2019). Applying the same approach for SARS-CoV-2, an open-label phase I trial was initiated to evaluate the safety, tolerability and immunogenicity of the INO-4800 DNA vaccine in 40 healthy volunteers (NCT04336410). Results are expected from the trial shortly and plans have materialized to start a phase II/III trial in the coming months (https://www.inovio.com/our-focus-serving-patients/covid-19/).

Currently, there are several RNA-based COVID-19 vaccine clinical trials in progress. For example, the positive results received from preclinical studies have encouraged the start of a phase I trial for an LNP mRNA SARS-CoV lead vaccine candidate, for which the German and Belgian authorities have provided regulatory approval (https://www.curevac.com/covid-19). Favorable results from the phase I study will lead to additional clinical trials with a significantly higher number of patients. In another approach, three COVID-19 vaccine candidates with uridine containing mRNA (uRNA) or nucleoside modified mRNA (modRNA) and one candidate based on saRNA are subjected to a randomized, placebo-controlled, observer-blind, dose-finding phase I/II trial (NCT04368728) in 200 healthy volunteers aged 18–55 years. The study will be assessed for safety and immunogenicity as well as the effects of repeated immunization. Preliminary results from the study (published on July 1, 2020) showed that immunization with the modRNA vaccine BNT162b1 at doses of 30 and 100 μg resulted in dose-related RBD-binding IgG concentrations and SARS-CoV-2 neutralizing titers (Mulligan et al., 2020). The geometric mean neutralizing titers were 1.8–2.8 fold higher than observed in a panel of COVID-19 convalescent human sera. After the LNP-encapsulated mRNA-1274 vaccine, which encodes the full-length, prefusion stabilized SARS-CoV-2 S protein, showed full protection against SARS-CoV-2 replication in lungs of mice (Corbett et al., 2020), an open-label, dose-ranging phase I trial was started in 150 healthy volunteers to validate the safety, reactogenicity and immunogenicity of the vaccine candidate (NTC04283461). The volunteers are subjected to a two-dose vaccination schedule 28 days apart, receiving 25, 50, 100, or 250 μg of LNP-mRNA intramuscularly. Initial evaluation of eight participants across the 25 and 100 μg cohorts indicated that the vaccination was generally safe and well-tolerated and elicited neutralizing antibody titers at the same level or higher compared to convalescent sera (https://investors.modernatx.com). Moreover, a randomized, observer-blind, placebo-controlled, dose-confirmation phase IIa clinical trial to evaluate the safety, reactogenicity and immunogenicity of the LNP-mRNA-1273 vaccine candidate is in progress (NCT04405076). In addition, the study protocol for a randomized, placebo-controlled phase III study has been recently finalized and the trial is expected to commence in July 2020 for 30,000 participants, applying an anticipated dose between 25 and 100 μg LNP-mRNA-1273 (https://investors.modernatx.com). Finally, a first-in-human randomized, placebo-controlled, observer-blind, dose-finding phase I/II clinical trial based on an LNP saRNA vector (Blakney et al., 2019) encoding the SARS-CoV-2 S gene has started in healthy volunteers (ISRCTN17072692). The study will initially include healthy volunteers aged 18–45 years but will later be expanded to 18–75 years old volunteers.

## Conclusions and Future Aspects

In summary, tremendous amounts of knowledge on the biology and molecular structure of SARS-CoV-2 has been gathered since the first reports of the COVID-19 outbreak at the end of 2019. However, the severity of the pandemic and the lack of antiviral drugs and vaccines has generated an unprecedented global search for solutions to conquer COVID-19 (Lundstrom, 2020). Although antiviral drug development is in full swing, the regional increase of new COVID-19 cases after the relaxation of lockdown and confinement conditions and the fear of a second wave of the pandemic, should further emphasize the importance of production of an efficient vaccine available for the global population. For this reason, there has been an accelerated effort on many fronts to reach this goal. No stones have been left unturned, including classic approaches such as the application of inactivated and live-attenuated viral particles. Numerous efforts to develop vaccines based on protein subunits and peptides, viral delivery vectors and nucleic acid-based vaccines using both DNA and RNA have been explored. Proof of principle of robust immune responses and protection against challenges with SARS-CoV-2 have been demonstrated for more or less all technologies mentioned above. The positive outcome from preclinical studies has further encouraged and accelerated the initiation of clinical trials, which in some cases already has reached the phase III level with positive outcome.

Understandably, much hype and speculations on if or when a functional vaccine will be available has put extra pressure on authorities and not the least on scientists. Too early and too optimistic interpretations of preliminary and often not peer-reviewed reports have misled the public and even experts in the field. Despite all this, we need to courageously move forward. The different approaches taken in parallel to reach the goal of obtaining a vaccine against COVID-19 should only be seen as a strength. Currently, there is no way to “declare a winner.” It is impossible to predict which technology will be successful and it cannot be stressed enough that all avenues should be explored in parallel. The COVID-19 vaccine field is moving at such a speed that even the most accelerated publication scheme cannot accurately keep up with the development, timely report the present situation and definitely not predict where we stand even in the near future. For this reason, an addendum had been included to provide a timely update of the present situation of COVID-19 vaccine development. However, as long as we all work together, we can also overcome the pandemic together.

## Addendum

The extremely rapid development of vaccines against COVID-19 since the submission of this review justifies an update of the current situation as of August 24, 2020. Today, 139 vaccine candidates have been subjected to preclinical studies in various animal models and 30 have reached different stages of clinical evaluation (https://www.who.int/publications/m/item/draft-landscape-of-covid-19-candidate-vaccines). Among the preclinical approaches, it is worth to mention that ministring DNA (msDNA) has been engineered to deliver VLPs against SARS-CoV-2 (www.mediphage.ca/applications-1). Immunization with msDNA VLPs elicited robust and long-lasting immunity against COVID-19 and also showed the potential of generating protection against other coronavirus infections.

In the context of clinical trials, interim analysis of phase I and II clinical trials with inactivated SARS-CoV-2 demonstrated common adverse reactions such as injection site pain and fever, but no serious adverse events (Xia et al., 2020). Furthermore, neutralizing antibodies against SARS-CoV-2 were detected in patients in both the phase I and II trials. Recruiting of patients from a healthy population of 18 years and older is in progress for a randomized, double-blind, parallel placebo-controlled phase III trial to evaluate the safety and protective efficacy (ChiCTR200034780). For another vaccine candidate based on inactivated SARS-CoV-2, recruiting has started for phase III trials in healthcare professionals in Brazil (NCT04456595) and in healthy volunteers in Indonesia (669/UN6.KEP/EC/2020).

The most controversial news for viral vector-based vaccine candidates relates to the approval of Sputnik V developed by the Gamaleya Research Institute of Epidemiology and Microbiology in Russia (Callaway, 2020). The adenovirus-based vaccine has only been evaluated in 76 volunteers in two early stage trials and has received much criticism for being approved before completion of any phase III trial. Moreover, results from neither preclinical nor clinical trials have been published. On a more positive note, the preliminary results on safety, reactogenicity and immunogenicity of a non-replicating chimpanzee adenovirus ChAdOx1-based COVID-19 vaccine in a phase I/II trial have been published (Folegatti et al., 2020). Healthy volunteers receiving a single intramuscular dose of 5 x 10^10^ viral particles showed no serious adverse events related to ChAdOx1 nCoV-19 and neutralizing antibody responses against SARS-CoV-2 were observed in 32 (91%) out of 35 individuals. A booster dose generated neutralizing antibodies including both humoral and cellular immune responses in all participants. These encouraging results supported large-scale vaccine evaluation in a phase III randomized, double-blind, placebo-controlled multicenter study in 30,000 adults (NCT04516746) with a starting date of August 17, 2020 and a primary completion date of December 2, 2020.

In the context of DNA-based COVID-19 vaccines, the safety, tolerability and immunogenicity of INO-4800 administered intradermally combined with electroporation has been evaluated in a phase I trial and has now entered phase II (NCT04447781). Similarly, the GX-19 COVID-19 vaccine candidate has reached phase II (NCT04445389). Finally, an LNP-encapsulated mRNA-based vaccine candidate showed no trial-limiting safety concerns in a phase I, dose-escalation, open-label trial including 45 healthy adults (Jackson et al., 2020). Vaccination with 25, 100 and 250 μg of LNP mRNA-1273 elicited higher antibody responses with the higher dose after the first immunization. The titers increased after the second immunization in all participants showing similar levels to convalescent serum specimens. Recruiting of 30,000 participants is now in progress for a randomized, stratified, observer-blind, placebo-controlled phase III trial (NCT04470427). Another LNP mRNA-based vaccine candidate, modRNA vaccine BNT162b1, which has shown positive results in a phase I trial (Mulligan et al., 2020), has also started recruiting more than 29,000 adults for a phase III trial (NCT04368728).

## Author Contributions

The author confirms being the sole contributor of this work and has approved it for publication.

## Conflict of Interest

The author acts as the CEO of the company PanTherapeutics without any financial benefits and the authoring of the review was conducted in the absence of any commercial or financial relationships that could be construed as a potential conflict of interest.

## Website

https://www.scienceboard.net/index.aspx?sec=sup&sub=Drug&pag=dis&ItemID=921

http://www.flowpharma.com

## References

[B1] Abdelmageed M. Abdelmoneim A. H. Mustafa M. I. Elfadol N. M. Murshed N. S. Shantier S. W.. (2020). Design of a multiepitope-based peptide vaccine against the E protein of human COVID-19: an immunoinformatics approach. Biomed. Res. Int. 2020:2683286. 10.1101/2020.02.04.934232 32461973 PMC7212276

[B2] Ahmad S. Zamry A. A. Tan H. T. Wong K. K. Lim J. Mohamud R. (2017). Targeting dendritic cells through gold particles: a review on the cellular uptake and subsequent immunological properties. Mol. Immunol. 91, 123–133. 10.1016/j.molimm.2017.09.001 28898717

[B3] Aleanizy F. S. Mohmed N. Alqahtani F. Y. Ali El Hadi Mohamed R. (2017). Outbreak of Middle east respiratory syndrome coronavirus in Saudi Arabia: a retrospective study. BMC Infect. Dis. 17:23. 10.1186/s12879-016-2137-3 28056850 PMC5217314

[B4] Anderson R. M. Fraser C. Ghani A. C. Donnelly C. A. Riley S. Ferguson N. M.. (2004). Epidemiology, transmission dynamics and control of SARS: the 2002–2003 epidemic. Philos. Trans. R. Soc. London B. Biol. Sci. 359, 1091–1105. 10.1098/rstb.2004.1490 15306395 PMC1693389

[B5] Armengaud J. Delaunay-Moisan A. Thuret J.-Y. van Anken E. Acosta-Alvear D. Arogon T.. (2020). The importance of naturally attenuated SARS-CoV-2 in the fight against COVID-19. Environ. Microbiol. 22, 1997–2000. 10.1111/1462-2920.15039 32342578 PMC7267670

[B6] Aves K.-L. Goksøyr L. Sander A. F. (2020). Advantages and prospects of Tag/Catcher mediated antigen display on Capsid-Like particle-based vaccines. Viruses 12:185. 10.3390/v12020185 32041299 PMC7077247

[B7] Azhar E. I. El-Kafrawy S. A. Farraj S. A. Hassan A. M. Al-Saeed M. S. Hashem A. M.. (2014). Evidence for camel-to-human transmission of MERS coronavirus. N. Engl. J. Med. 370, 2499–2505. 10.1056/NEJMoa1401505 24896817

[B8] Baker M. P. Reynolds H. M. Lumicisi B. Bryson C. J. (2010). Immunogenicity of protein therapeutics: The key causes, consequences and challenges. Self Nonself 1, 314–322. 10.4161/self.1.4.13904 21487506 PMC3062386

[B9] Barhate G. Gautam M. Gairola S. Jadhav S. Pokharkar V. (2014). Enhanced mucosal immune responses against tetanus toxoid using novel delivery system comprised of chitosan-functionalized gold nanoparticles and botanical adjuvant: characterization, immunogenicity, and stability assessment. J. Pharm. Sci. 103, 3448–3456. 10.1002/jps.24161 25219511

[B10] Barrett P. N. Terpening S. J. Snow D. Cobb R. R. Kistner O. (2017). Vero cell technology for rapid development of inactivated whole virus vaccines for emerging viral diseases. Expert Rev. Vaccines 16, 883–894. 10.1080/14760584.2017.1357471 28724343

[B11] Bashirullah A. Cooperstock R. L. Lipshitz H. D. (2001). Spatial and temporal control of RNA stability. Proc. Natl. Acad. Sci. U.S.A. 98, 7025–7028. 10.1073/pnas.111145698 11416182 PMC34617

[B12] Bernstein P. Peltz S. W. Ross J. (1989). The poly(A)-poly(A)-binding protein complex is a major determinant of mRNA stability *in vitro*. Mol. Cell. Biol. 9, 659–670. 10.1128/MCB.9.2.659 2565532 PMC362643

[B13] Bittle J. L. Houghton R. A. Alexander H. Shinnick T. M. Sutcliffe J. G. Lerner R. A.. (1982). Protection against foot and mouth disease by immunisation with a chemically synthesised peptide predicted from the viral nucleotide sequence. Nature 298, 30–33. 10.1038/298030a0 7045684

[B14] Blakney A. K. McKay P. F. Yus B. I. Aldon Y. Shattock R. J. (2019). Inside out: optimization of lipid nanoparticle formulations for exterior complexation and *in vivo* delivery of saRNA. Gene Ther. 26, 363–372. 10.1038/s41434-019-0095-2 31300730 PMC6760535

[B15] Bradburne A. F. Bynoe M. L. Tyrell D. A. J. (1967). Effects of a “new” human respiratory virus in volunteers. Br. Med. J. 3, 767–769. 10.1136/bmj.3.5568.767 6043624 PMC1843247

[B16] Brandler S. Ruffié C. Combridet C. Brault J. B. Najburg V. Prevost M. C.. (2013). A Recombinant measles vaccine expressing Chikungunya Virus-like particles is strongly immunogenic and protects mice from lethal challenge with Chikungunya virus. Vaccine 31, 3718–3725. 10.1016/j.vaccine.2013.05.086 23742993

[B17] Brawerman G. (1974). Eukaryotic messenger RNA. Annu. Rev. Biochem. 43, 621–642. 10.1146/annurev.bi.43.070174.003201 4604447

[B18] Broekhuijsen M.P. Blom T. van Rijn J. Pouwels P.H. Klasen E.A. Fasbender M.J.. (1986). Synthesis of fusion proteins with multiple copies of antigenic determinant of foot-and-mouth disease virus. Gene 49, 189–197. 10.1016/0378-1119(86)90279-9 2436976

[B19] Burgess D. J. (2012). RNA stability: remember your driver. Nat. Rev. Genet. 13:72. 10.1038/nrg3157 22230816

[B20] Callaway E. (2020). Russia's fast-track coronavirus vaccine draws outrage over safety. Nature 584, 334–335. 10.1038/d41586-020-02386-2 32782400

[B21] Chen W. H. Du L. Chag S. M. Ma C. Tricoche N. Tao X.. (2014). Yeast-expressed recombinant protein of the receptor-binding domain in SARS-CoV spike protein with deglycosylated forms as a SARS vaccine candidate. Hum. Vaccin. Immunother. 10, 648–658. 10.4161/hv.27464 24355931 PMC4130269

[B22] Chen W. H. Hotez P. J. Bottazzi M. E. (2020). Potential for developing a SARS-CoV Receptor Binding Domain (RBD) recombinant protein as a heterologous human vaccine against Coronavirus Infectious Disease (COVID-19). Hum. Vacc. Immunother. 16, 1239–1242. 10.1080/21645515.2020.1740560 32298218 PMC7482854

[B23] Cheng B. Y. H. Nogales A. de la Torre J. C. Martinez-Sobrido L. (2017). Development of live-attenuated arenavirus vaccines based on codon deoptimization of the viral glycoprotein. Virology 501, 35–46. 10.1016/j.virol.2016.11.001 27855284 PMC5201438

[B24] Cherry J. D. (2004). The chronology of the 2002–2003 SARS mini pandemic. Paediatr. Resp. Rev. 5, 262–269. 10.1016/j.prrv.2004.07.009 15531249 PMC7106085

[B25] Clarke B. E. Newton S. E. Carroll A. R. Francis M. J. Appleyard G. Syred A. D.. (1987). Improved immunogenicity of a peptide epitope after fusion to hepatitis B core protein. Nature 330, 381–384. 10.1038/330381a0 2446137

[B26] Conry R. M. LoBuglio A. F. Wright M. Sumerel L. Pike M. J. Johanning F.. (1995). Characterization of a messenger RNA polynucleotide vaccine vector. Cancer Res. 55, 1397–400. 7882341

[B27] Corbett K. S. Edwards D. Leist S. R. Abiona O. M. Boyoglu-Barnum S. Gillispie R. A.. (2020). SARS-CoV-2 mRNA vaccine development enabled by prototype pathogen preparedness. bioRxiv. 2020:145920. 10.1101/2020.06.11.145920 32756549 PMC7581537

[B28] Criscuolo E. Caputo V. Diotti R. A. Sautto G. A. Kirchenbaum G. A. Clementi N. (2019). Alternative methods of vaccine delivery: An overview of edible and intradermal vaccines. J. Immunol. Res. 2019:8303648. 10.1155/2019/8303648 30949518 PMC6425294

[B29] Darquet A. M. Cameron B. Wils P. Scherman D. Crouzet J. (1997). A new DNA vehicle for nonviral gene delivery: supercoiled minicircle. Gene Ther. 4, 1341–1349. 10.1038/sj.gt.3300540 9472558

[B30] Deans T. L. Singh A. Gibson M. Elisseeff J. H. (2012). Regulating synthetic gene networks in 3D materials. Proc. Natl. Acad. Sci. U.S.A. 109, 15217–15222. 10.1073/pnas.1204705109 22927376 PMC3458344

[B31] Delrue I. Verzele D. Madder A. Nauwynck H. J. (2012). Inactivated virus vaccines from chemistry to prophylaxis: Merits, risks and challenges. Expert Rev. Vaccines 11, 695–719. 10.1586/erv.12.38 22873127

[B32] Dileo J. Miller T. E. Jr Chesnoy S. Huang L. (2003). Gene transfer to subdermal tissues via a new gene gun design. Hum. Gene Ther. 14, 79–87. 10.1089/10430340360464732 12573061

[B33] Ding C. Ma J. Dong Q. Liu Q. (2018). Live bacterial vaccine vector and delivery strategies of heterologous antigen: a review. Immunol. Lett. 197, 70–77. 10.1016/j.imlet.2018.03.006 29550258

[B34] Domi A. Feldmann F. Basu R. McCurley N. Schifflett K. Emanuel J.. (2018). A single dose of modified vaccinia ankara expressing ebola virus like particles protects non-human primates from lethal ebola virus challenge. Sci Rep. 8:864. 10.1038/s41598-017-19041-y 29339750 PMC5770434

[B35] Eckerle I. Corman V. M. Muller M. A. Lenk M. Ulrich R. G. Drosten C. (2014). Replicative capacity of MERS Coronavirus in livestock cell lines. Emerg. Infect. Dis. 20, 276–279. 10.3201/eid2002.131182 24457147 PMC3901466

[B36] Folegatti P. M. Bellamy D. Roberts R. Powlson J. Edwards N. J. Mair C. F.. (2019). Safety and immunogenicity of a novel recombinant simian Adenovirus ChAdOx2 as a vectored vaccine. Vaccines 7:40. 10.3390/vaccines7020040 31096710 PMC6630572

[B37] Folegatti P. M. Ewer K. J. Aley P. K. Angus B. Becker S. Belij-Rammerstorfer S.. (2020). Safety and immunogenicity of the ChAdOx1 nCoV-19 vaccine against SARS-CoV-2: a preliminary report of a phase 1/2, single-blind, randomized controlled trial. Lancet 396, 467–478. 10.1016/S0140-6736(20)31604-4 32702298 PMC7445431

[B38] Francis M. J. (2018). Recent advances in vaccine technologies. Vet. Clin. Small Anim. 48, 231–241. 10.1016/j.cvsm.2017.10.002 29217317 PMC7132473

[B39] Francis M. J. Hastings G. Z. Syred A. D. McGinn B. Brown F. Rowlands D. J. (1987). Non-responsiveness to a foot-and mouth disease virus peptide overcome by addition of foreign helper T-cell determinants. Nature 330, 168–170. 10.1038/330168a0 2444892

[B40] Fukuhara H. Ino Y. Todo T. (2016). Oncolytic virus therapy: a new era of cancer therapy at dawn. Cancer Sci. 107, 1373–1379. 10.1111/cas.13027 27486853 PMC5084676

[B41] Gao Q. Bao L. Mao H. Wang L. Xu K. Yang M.. (2020). Development of an inactivated vaccine candidate for SARS-CoV-2. Science 369, 77–81. 10.1126/science.abc1932 32376603 PMC7202686

[B42] Grantham R. Gautier C. Gouy M. Mercier R. Pave A. (1980). Codon catalog usage and the genome hypothesis. Nucl. Acids Res. 8, r49–r62. 10.1093/nar/8.1.197-c 6986610 PMC327256

[B43] Güthe S. Kapinos L. Möglich A. Meier S. Grzesiek S. Kiefhaber T. (2004). Very fast folding and association of a trimerization domain from bacteriophage T4 fibritin. J. Mol. Biol. 337, 905–915. 10.1016/j.jmb.2004.02.020 15033360

[B44] Hamre D. Procknow J. J. (1966). A new virus isolated from the human respiratory tract. Proc. Soc. Exp. Biol. Med. 121, 190–193. 10.3181/00379727-121-30734 4285768

[B45] Harbury P. B. Kim P. S. Alber T. (1994). Crystal structure of an isoleucine-zippertrimer. Nature 371, 80–83. 10.1038/371080a0 8072533

[B46] Herst C. V. Burkholz S. Sidney J. Sette A. Harris P. E. Massey S.. (2020). An effective CTL peptide vaccine for Ebola Zaire based on survivors' CD8+ targeting of a particular nucleocapsid protein epitope with potential implications for COVID-19 vaccine design. Vaccine 38, 4464–4475. 10.1016/j.vaccine.2020.04.034 32418793 PMC7186210

[B47] Hess P. R. Boczkowski D. Nair S. K. Snyder D. Gilboa E. (2006). Vaccination with mRNAs encoding tumor-associated antigens and granulocyte-macrophage colony-stimulating factor efficiently primes CTL responses, but is insufficient to overcome tolerance to a model tumor/self antigen. Cancer Immunol. Immunother. 55, 672–683. 10.1007/s00262-005-0064-z 16133108 PMC11030883

[B48] Hoerr I. Obst R. Rammensee H. G. Jung G. (2000). *In vivo* application of RNA leads to induction of specific cytotoxic T lymphocytes and antibodies. Eur. J. Immunol. 30, 1–7. 10.1002/1521-4141(200001)30:1<1::AID-IMMU1>3.0.CO;2-# 10602021

[B49] Holtkamp S. Kreiter S. Selmi A. Simon P. Koslowski M. Huber C.. (2006). Modification of antigen-encoding RNA increases stability, translational efficacy, and T cell stimulatory capacity of dendritic cells. Blood 108, 4009–4017. 10.1182/blood-2006-04-015024 16940422

[B50] Hooper J. W. Moon J. E. Paolino K. M. Newcomer R. McLain D. E. Josleyn M.. (2014). A phase 1 clinical trial of Hantaan virus and Puumala virus M-segment DNA vaccines for haemorrhagic fever with renal syndrome delivered by intramuscular electroporation. Clin. Microbiol. Infect. 20, 110–117. 10.1111/1469-0691.12553 24447183

[B51] Houseley J. Tollervey D. (2009). The many pathways of RNA degradation. Cell 136, 763–776. 10.1016/j.cell.2009.01.019 19239894

[B52] Huang M. Chen Z. Hu S. Jia F. Li Z. Hoyt G.. (2009). Novel minicircle vector for gene therapy in murine myocardial infarction. Circulation 120, S230–S237. 10.1161/CIRCULATIONAHA.108.841155 19752373 PMC3163107

[B53] Huo J. Le Bas A. Ruza R. R. Duyvesteyn H. M. E. Mikolajek H. Malinauskas T.. (2020). Neutralizing nanobodies bind SARS-CoV-2 spike RBD and block interaction with ACE2. Nat. Struct. Mol. Biol. 27, 846–854. 10.1038/s41594-020-0469-6 32661423

[B54] Jackson L. A. Anderson E. J. Rouphael N. G. Roberts P. C. Makhene M. Coler R. N.. (2020). An mRNA vaccineagainst SARS-CoV-2 – preliminary report. N. Engl. J. Med. 10.1056/NEJMoa2022483. [Epub ahead of print]. PMC7377258 32663912

[B55] Jazayeri S. D. Poh C. L. (2019). Recent advances in delivery of veterinary DNA vaccines against avian pathogens. Veterinary Res. 50:78. 10.1186/s13567-019-0698-z 31601266 PMC6785882

[B56] Kaplan E. H. (2020). Containing 2019-nCoV (Wuhan) coronavirus. Health Care Manag. Sci. 23, 311–314. 10.1007/s10729-020-09504-6 32146554 PMC7087552

[B57] Kariko K. Buckstein M. Ni H. Weissman D. (2005). Suppression of RNA recognition by Toll-like receptors: the impact of nucleoside modification and the evolutionary origin of RNA. Immunity 23, 165–175. 10.1016/j.immuni.2005.06.008 16111635

[B58] Kariko K. Muramatsu H. Ludwig J. Weissman D. (2011). Generating the optimal mRNA for therapy: HPLC purification eliminates immune activation and improves translation of nucleoside-modified, protein-encoding mRNA. Nucl. Acids Res. 39:e142. 10.1093/nar/gkr695 21890902 PMC3241667

[B59] Kariko K. Muramatsu H. Welsh F. A. Ludwig J. Kato H. Akira S.. (2008). Incorporation of pseudouridine intom, RN. A., yields superior nonimmunogenic vector with increased translational capacity and biological stability. Mol.Ther. 16, 1833–40. 10.1038/mt.2008.200 18797453 PMC2775451

[B60] Kim E. Erdos G. Huang S. Kenniston T. W. Balmert S. C. Donahue Carey C.. (2020). Microneedle array delivered recombinant Coronavirus vaccines: Immunogenicity and rapid translational development. EBio Med. 55:102743. 10.1016/j.ebiom.2020.102743 32249203 PMC7128973

[B61] Kim T. J. Jin H.-T. Hur S.-Y. Yang H. G. Seo Y. B. Hong S. R.. (2014). Clearance of persistent HPV infection and cervical lesion by therapeutic DNA vaccine in CIN3 patients. Nat. Commun. 5:5317. 10.1038/ncomms6317 25354725 PMC4220493

[B62] Kose N. Fox J. M. Sapparapu G. Bombardi R. Tennekoon R. N. Dharshan da Silva A.. (2020). A lipid-encapsulated mRNA encoding a potently neutralizing human monoclonal antibody protects against chikungunya infection. Sci. Immunol. 4:eaaw6647 10.1126/sciimmunol.aaw6647 31101672 PMC6629435

[B63] Lai C. C. Liu Y. H. Wang C. Y. Wang Y. H. Hsueh S. C. Yen M. Y.. (2020). Asymptomatic carrier state, acute respiratory disease, and pneumonia due to severe acute respiratory syndrome coronavirus 2 (SARS-CoV-2): facts and myths. J. Microbiol. Immunol. Infect. 53, 404–412. 10.1016/j.jmii.2020.02.012 32173241 PMC7128959

[B64] Lee J. Kumar S. A. Jhan Y. Y. Bishop C. J. (2018). Engineering DNA vaccines against infectious diseases. Acta Biomater. 80, 31–47. 10.1016/j.actbio.2018.08.033 30172933 PMC7105045

[B65] Li K. Li Z. Wohlford-Lenane C. Meyerholz D. K. Channappanavar R. An D.. (2020). Single-dose, intranasal immunization with recombinant parainfluenza virus 5 expressing Middle East Respiratory Syndrome Coronavirus (MERS-CoV) Spike protein protects mice from fatal MERS-CoV infection. mBio 11:e00554–20. 10.1128/mBio.00554-20 32265331 PMC7157776

[B66] Li W. Moore M. J. Vasilieva N. Sui J. Wong S. K. Berne M. A.. (2003). Angiotensin-converting enzyme 2 is a functional receptor for the SARS coronavirus. Nature 426, 450–454. 10.1038/nature02145 14647384 PMC7095016

[B67] Liu M. A. Ulmer J. B. (2005). Human clinical trials of plasmid DNA vaccines. Adv. Genet. 55, 25–40. 10.1016/S0065-2660(05)55002-8 16291211

[B68] Lundstrom K. (2017). Latest trends in cancer gene therapy applying viral vectors. Fut. Virol. 12, 667–684. 10.2217/fvl-2017-0070

[B69] Lundstrom K. (2018). Latest development on RNA-based drugs and vaccines. Future Sci. OA 4:FSO300. 10.4155/fsoa-2017-0151 29796303 PMC5961404

[B70] Lundstrom K. (2019). Self-amplifying RNA virus vectors: clinical applications in cancer drug discovery. Exp. Opin. Drug Discov. 16, 1027–1029. 10.1080/17425247.2019.1653851 31387406

[B71] Lundstrom K. (2020). Coronavirus pandemic – therapy and vaccines. Biomedicines 8:E109. 10.3390/biomedicines8050109 32375268 PMC7277397

[B72] Lundstrom K. Boulikas T. (2003). Viral and non-viral vectors in gene therapy. technology development and clinical trials. Technol. Cancer Res. Treat. 2, 471–486. 10.1177/153303460300200513 14529313

[B73] McBurney S. P. Sunshine J. E. Gabriel S. Huynh J. P. Sutton W. F. Fuller D. H.. (2016). Evaluation of protection induced by a dengue virus serotype 2 envelope domain III protein scaffold/DNA vaccine in non-human primates. Vaccine 34, 3500–3507. 10.1016/j.vaccine.2016.03.108 27085173 PMC4959041

[B74] McNamara M. A. Nair S. K. Holl E. K. (2015) RNA-based vaccines in cancer immunotherapy. J. Immunol. Res. 2015:794528. 10.1155/2015/794528 26665011 PMC4668311

[B75] Meyer B. Muller M. A. Corman V. M. Al-Masri M. Hossain R. Madani H.. (2014). Antibodies against MERS coronavirus in dromedary camels, United Arab Emirates, 2003 and 2013. Emerg. Infect. Dis. 20, 552–559. 10.3201/eid2004.131746 24655412 PMC3966379

[B76] Minor P. D. (2015). Live attenuated vaccines: Historical successes and current challenges. Virology 479-480, 379–392. 10.1016/j.virol.2015.03.032 25864107

[B77] Mockey M. Gonçalves C. Dupuy F. P. Lemoine F. M. Pichon C. Midoux P. (2006). mRNA transfection of dendritic cells: synergistic effect of ARCA mRNA capping with Poly(A) chains in cis and in trans for a high protein expression level. Biochem. Biophys. Res. Comm. 340, 1062–1068. 10.1016/j.bbrc.2005.12.105 16403444

[B78] Modjarrad K. Roberts C. C. Mills K. T. Castellano A. R. Paolino K. Muthumani K.. (2019). Safety and immunogenicity of an anti-middle east respiratory syndrome Coronavirus DNA vaccine: a phase I, open-label, single-arm, dose-escalation trial. Lancet Infect. Dis. 19, 1013–1022. 10.1016/S1473-3099(19)30266-X 31351922 PMC7185789

[B79] Morein B. Sharp M. Sundquist B. Simons K. (1983). Protein subunit vaccines of Parainfluenza Type 3 virus: Immunogenic effect in lambs and mice. J. Gen. Virol. 64, 1557–1569. 10.1099/0022-1317-64-7-1557 6306152

[B80] Morris A. E. Remmele R. L. Jr Klinke R. Macduff B. M. Fanslow W. C. Armitage R. J. (1999). Incorporation of an isoleucine zipper motif enhances the biological activity of soluble CD40L(CD154). J. Biol. Chem. 274, 418–423. 10.1074/jbc.274.1.418 9867859

[B81] Morse M. A. Nair S. K. Mosca P. J. Hobeika A. C. Clay T. M. Deng Y.. (2003). Immunotherapy with autologous, human dendritic cells transfected with carcinoembryonic antigen mRNA. Cancer Invest. 21, 341–349. 10.1081/CNV-120018224 12901279

[B82] Mulligan M. J. Lyke K. E. Kitchin N. Absalon J. Gurtman A. Lockhart S.. (2020). Phase 1/2 study of a COVID-19 RNA vaccine BNT162b1. Nature 10.1038/s41586-020-2639-4. [Epub ahead of print]. 32785213

[B83] Munroe D. Jacobson A. (1990). mRNA Poly(A) tail, a 3' enhancer of translational initiation. Mol. Cell. Biol. 10, 3441–3455. 10.1128/MCB.10.7.3441 1972543 PMC360780

[B84] Mutua G. Anzala O. Luhn K. Robinson C. Bockstal V. Anumendem D.. (2019). Safety and immunogenicity of a 2-dose heterologous vaccine regimen with Ad26.ZEBOV and MVA-BN-Filo Ebola vaccines: 12-month data from a Phase 1 randomized clinical trial in Nairobi, Kenya. (2019). J. Infect. Dis. 220, 57–67. 10.1093/infdis/jiz071 30796816 PMC6548899

[B85] Nagata T. Uchijima M. Yoshida A. Kawashima M. Koide Y. (1999). Codon optimization effect on translational efficiency of DNA vaccine in mammalian cells: analysis of plasmid DNA encoding a CTL epitope derived from microorganisms. Biochem. Biophys. Res. Commun. 261, 445–451. 10.1006/bbrc.1999.1050 10425204

[B86] Ng T. W. Turinici G. Danchin A. (2003). A double epidemic model for the SARS propagation. BMC Infect. Dis. 3:19. 10.1186/1471-2334-3-19 12964944 PMC222908

[B87] Nilsson C. Hejdeman B. Godoy-Ramirez K. Tecleab T. Scarlatti G. Bråve A.. (2015). HIV-DNA given with or without intradermal electroporation is safe and highly immunogenic in healthy Swedish HIV-1 DNA/MVA vaccinees: a phase I randomized trial. PLoS ONE 10:e0131748. 10.1371/journal.pone.0131748 26121679 PMC4486388

[B88] Pardi N. Weissman D. (2017). Measuring the adjuvant activity of RNA vaccines. Methods Mol. Biol. 1499, 143–153. 10.1007/978-1-4939-6481-9_8 27987147

[B89] Pascarella G. Strumia A. Piliego C. Bruno F. Del Buono R. Costa F.. (2020). COVID-19 diagnosis and management: a comprehensive review. J. Intern. Med. 288, 192–206. 10.1111/joim.13091 32348588 PMC7267177

[B90] Peiris J. S. Yuen K. Y. Osterhaus A. D. Stöhr K. (2003). The severe acute respiratory syndrome. N. Engl. J. Med. 349, 2431–2441. 10.1056/NEJMra032498 14681510

[B91] Perche F. Benvegnu T. Berchel M. Lebegue L. Pichon C. Jaffrès P. A.. (2011). Enhancement of dendritic cells transfection *in vivo* and of vaccination against B16F10 melanoma with mannosylated histidylated lipopolyplexes loaded with tumor antigen messenger RNA. Nanomedicine 7, 445–53. 10.1016/j.nano.2010.12.010 21220051

[B92] Phua K. K. L. Leong K. W. Nair S. K. (2013). Transfection efficiency and transgene expression kinetics of mRNA delivered in naked and nanoparticle format. J. Contr. Release 166, 227–33. 10.1016/j.jconrel.2012.12.029 23306021 PMC3594075

[B93] Probst J. Brechtel S. Scheel B. Hoerr I. Jung G. Rammensee H. G.. (2006). Characterization of the ribonuclease activity on the skin surface. Genetic Vacc. Ther. 4:4. 10.1186/1479-0556-4-4 16732888 PMC1524753

[B94] Reisinger E. C. Tschismarov R. Beubler E. Wiedermann U. Firbas C. Loebermann M.. (2019). Immunogenicity, safety and tolerability of the measles-vectored chikungunya virus vaccine MV-CHIK: a double-blind, randomised, placebo-controlled and active-controlled phase 2 trial. Lancet 392, 2718–2727. 10.1016/S0140-6736(18)32488-7 30409443

[B95] Rubsamen R. M. Herst C. V. Lloyd P. M. Heckerman D. E. (2014). Eliciting cytotoxic T-lymphocyte responses from synthetic vectors containing one or two epitopes in a C57BL/6 mouse model using peptide-containing biodegradable microspheres and adjuvants. Vaccine 32, 4111–4116. 10.1016/j.vaccine.2014.05.071 24912025

[B96] Salvato M. S. Domi A. Guzman-Cardazo Modina-Moreno S. Zapata J. C. Hsu H.. (2018). A single dose of modified vaccinia ankara expressing lassa virus-like particles protects mice from lethal intra-cerebral virus challenge. Pathogens 8:133. 10.3390/pathogens8030133 31466243 PMC6789566

[B97] Schlake T. Thess A. Fotin-Mleczek M. Kallen K.-J. (2012). Developing mRNA-vaccine technologies. RNA Biol. 9, 1319–1330. 10.4161/rna.22269 23064118 PMC3597572

[B98] Shen X. Pitol A. K. Bachmann V. Hacker D. L. Baldi L. Wurm F. M. (2015). A simple plasmid-based transient gene expression method using high five cells. J. Biotechnol. 216, 67–75. 10.1016/j.jbiotec.2015.10.007 26476358

[B99] Sköld A. E. van Beek J. J. Sittig S. P. Bakdash G. Tel J. Schreibelt G.. (2015). Protamine-stabilized RNA as an ex vivo stimulant of primary human dendritic cell subsets. Cancer Immunol. Immunother. 64, 1461–1473. 10.1007/s00262-015-1746-9 26275446 PMC4612318

[B100] Sliepen K. van Montfort T. Melchers M. Isik G. Sanders R. W. (2015). Immunosilencing a highly immunogenic protein trimerization domain. J. Biol. Chem. 290, 7436–7442. 10.1074/jbc.M114.620534 25635058 PMC4367253

[B101] Smith T. R. F. Patel A. Ramos S. Elwood D. Zhu X. Yan J.. (2020). Immunogenicity of a DNA vaccine candidate for COVID-19. Nat. Commun. 11:2601. 10.1038/s41467-020-16505-0 32433465 PMC7239918

[B102] Stachyra A. Redkiewicz P. Kosson P. Protasiuk A. Gora-Sochacka A. Kudla G. (2016). Codon optimization of antigen coding sequences improves the immune potential of DNA vaccines against avian influenza virus H5N1 in mice and chickens. Virol. J. 13:143. 10.1186/s12985-016-0599-y 27562235 PMC5000471

[B103] Stefano M. L. Kream R. M. Stefano G. B. (2020). A novel vaccine employing non-replicating rabies virus expressing chimeric SARS-CoV-2 Spike protein domains: functional inhibition of viral/nicotinic acetylcholine receptor complexes. Med. Sci. Monit. 26:e926016. 10.12659/MSM.926016 32463026 PMC7278327

[B104] Steitz J. Britten C. M. Wolfel T. Tuting T. (2006). Effective induction of anti-melanoma immunity following genetic vaccination with synthetic mRNA coding for the fusion protein EGFP.TRP2. Cancer Immunol. Immunother. 55, 246–253. 10.1007/s00262-005-0042-5 16133114 PMC11030217

[B105] Stepinski J. Waddell C. Stolarski R. Darzynkiewicz E. Rhoads R. E. (2001). Synthesis and properties of mRNAs containing the novel ‘anti-reverse' cap analogs 7-methyl (3'-O-methyl) GpppG and 7-methyl (33'-deoxy) GpppG, RNA 7, 1486–1495. 11680853 PMC1370192

[B106] Sun J. Li D. Hao Y. Zhang Y. Fan W. Fu J.. (2009). Posttranscriptional regulatory elements enhance antigen expression and DNA vaccine efficacy. DNA Cell Biol. 28, 233–240. 10.1089/dna.2009.0862 19388846 PMC2719066

[B107] Sunshine J. C. Bishop C. J. Green J. J. (2011). Advances in polymeric and inorganic vectors for nonviral nucleic acid delivery. Ther. Deliv. 2, 493–521. 10.4155/tde.11.14 22826857 PMC4000586

[B108] Thompson M. Heath S. L. Sweeton B. Williams K. Cunningham P. Keele B. F.. (2016). DNA/MVA vaccination of HIV-1 infected participants with viral suppression on antiretroviral therapy, followed by treatment interruption: elicitation of immune responses without control of re-emergent virus. PLoS ONE 11:e0163164. 10.1371/journal.pone.0163164 27711228 PMC5053438

[B109] Tlaxca J. Ellis S. Remmele R. L. Jr. (2015) Live attenuated inactivated viral vaccine formulation nasal delivery: potential challenges. Adv. Drug Deliv. Rev. 93 56–78. 10.1016/j.addr.2014.10.002 25312673

[B110] Torrey H. L. Kaliaperumal V. Bramhecha Y. Weir G. M. Falsey A. R. Walsh E. E.. (2020). Evaluation of the prospective potential of antibody and T cell responses elicited by a novel preventative vaccine towards respiratory syncytial virus small hydrophobic protein. Hum. Vaccin. Immunother. 10.1080/21645515.2020.1756671. [Epub ahead of print]. PMC7553696 32530723

[B111] Tsunetsugu-Yokota Y. (2008). Large-scale preparation of UV-inactivated SARS Coronavirus virions for vaccine antigen. Methods Mol. Biol. 454, 119–126. 10.1007/978-1-59745-181-9_11 19057880 PMC7122600

[B112] van der Velden A. W. Thomas A. A. (1999). The role of the 5' untranslated region of an mRNA in translation regulation during development. Int. J. Biochem. Cell Biol. 31, 87–106. 10.1016/S1357-2725(98)00134-4 10216946

[B113] van Doremalen N. Lambe T. Spencer A. Belij-Rammerstorfer S. Purushotham J. N. Port J. R.. (2020). ChAdOx1 nCov-19 vaccination prevents SARS-CoV-2 pneumonia in rhesus macaques. bioRxiv. 2020:093195. 10.1101/2020.05.13.093195 33469217

[B114] van Doremalen N. Miazgowicz K. L. Milne-Price S. Bushmaker T. Robertson S. Scott D.. (2014). Host species restriction of Middle East respiratory syndrome coronavirus through its receptor dipeptidyl peptidase 4. J. Virol. 88, 9220–9232. 10.1128/JVI.00676-14 24899185 PMC4136254

[B115] Vitelli A. Folgori A. Scarselli E. Colloca S. Capone S. Nicosia A. (2017). Chimpanzee adenoviral vectors as vaccines – challenges to move the technology into the fast lane. Expert Rev. Vaccines 16, 1241–1252. 10.1080/14760584.2017.1394842 29047309

[B116] Wang H. Zhang Y. Huang B. Deng W. Quan Y. Wang W.. (2020). Development of an inactivated vaccine candidate, BBIBP-CorV, with potent protection against SARS-CoV-2. Cell 182, 713–721.e9. 10.1016/j.cell.2020.06.008 32778225 PMC7275151

[B117] Wolff J. A. Malone R. W. Williams P. Chong W. Acsadi G. Jani A.. (1990). Direct gene transfer into mouse muscle *in vivo*. Science 247(4949 Pt 1), 1465–1468. 10.1126/science.1690918 1690918

[B118] Xia S. Duan K. Zhang Y. Zhao D. Zhang H. Xie Z.. (2020). Effect of an inactivated vaccine against SARS-CoV-2 on Safety and Immunogenicity Outcomes. interim analysis of 2 randomized clinical trials. JAMA 324, 1–10. 10.1001/jama.2020.15543 32789505 PMC7426884

[B119] Xiao X. Feng Y. Chakraborti S. Dimitrov D. S. (2004). Oligomerization of the SARS-CoV S glycoprotein: Dimerization of the N-terminus and trimerization of the ectodomain. Biochem. Biophys. Res. Commun. 322, 93–99. 10.1016/j.bbrc.2004.07.084 15313178 PMC7092807

[B120] Xu Y. Liang W. Qiu Y. Cespi M. Palmieri G. F. Mason A. J.. (2016). Incorporation of a nuclear localization signal in pH responsive LAH4-L1 peptide enhances transfection and nuclear uptake of plasmid DNA. Mol. Pharm. 13, 3141–3152. 10.1021/acs.molpharmaceut.6b00338 27458925

[B121] Yang Y. Peng F. Wang R. Yange M. Guan K. Jiang T.. (2020). The deadly coronavirus; The 2003 SARS epidemic and the 2020 novel coronavirus epidemic in China. J. Autoimmun. 109:102434. 10.1016/j.jaut.2020.102434 32143990 PMC7126544

[B122] Yu J. Tostanoski L. H. Peter L. Mercado N. B. McMahan K. Mahrokhian S. H.. (2020). DNA vaccine protection against SARS.CoV-2 in rhesus macaques. Science. 369, 806–811. 10.1126/science.abc6284 32434945 PMC7243363

[B123] Zaki A. M. van Boheemen S. Bestebroer T. M. Osterhaus A. D. Fouchier R. A. (2012). Isolation of a novel coronavirus from a man with pneumonia in Saudi Arabia. N. Engl. J.Med. 367, 1814–1820. 10.1056/NEJMoa1211721 23075143

[B124] Zheng Z. Diaz-Arévalo D. Guan H. Zeng M. (2018) Noninvasive vaccination against infectious diseases. Hum. Vaccine Immunother. 14, 1717–1733. 10.1080/21645515.2018.1461296 29624470 PMC6067898

[B125] Zhou Z. Post P. Chubet R. Holtz K. McPherson C. Petric M.. (2006). A recombinant baculovirus-expressed S glycoprotein vaccine elicits high titers of SARS-associated coronavirus (SARS-CoV) neutralizing antibodies in mice. Vaccine 24, 3624–3631. 10.1016/j.vaccine.2006.01.059 16497416 PMC7115485

[B126] Zhu F.-C. Li Y.-H. Guan X.-H. Hou L.-H. Wang W.-J. Li J.-X.. (2020). Safety, tolerability, and immunogenicity of a recombinant adenovirus type-5 vectored COVID-19 vaccine: a dose-escalation, open-label, non-randomised, first-in-human trial. Lancet 395, 1845–1854. 10.1016/S0140-6736(20)31208-3 32450106 PMC7255193

[B127] Zohra F. T. Chowdhury E. H. Tada S. Hoshiba T. Akaike T. (2007). Effective delivery with enhanced translational activity synergistically accelerates mRNA-based transfection. Biochem. Biophys. Res. Comm. 358, 373–378. 10.1016/j.bbrc.2007.04.059 17475211

